# 3D printed hydrogel scaffolds combining glutathione depletion-induced ferroptosis and photothermia-augmented chemodynamic therapy for efficiently inhibiting postoperative tumor recurrence

**DOI:** 10.1186/s12951-022-01454-1

**Published:** 2022-06-07

**Authors:** Wentao Dang, Wei-Chih Chen, Enguo Ju, Yanteng Xu, Kai Li, Haixia Wang, Kun Wang, Shixian Lv, Dan Shao, Yu Tao, Mingqiang Li

**Affiliations:** 1 Laboratory of Biomaterials and Translational Medicine, Center for Nanomedicine, The Third Affiliated Hospital, Sun Yat-Sen University, Guangzhou, 510630 China; 2 Department of Joint and Trauma Surgery, The Third Affiliated Hospital, Sun Yat-Sen University, Guangzhou, 510630 China; 3 Department of Ultrasound, The Third Affiliated Hospital, Sun Yat-Sen University, Guangzhou, 510630 China; 4 School of Materials Science and Engineering, Peking University, Beijing, 100871 China; 5 Institutes of Life Sciences, School of Biomedical Sciences and Engineering, South China University of Technology, Guangzhou, 510006 China; 6 Guangdong Provincial Key Laboratory of Liver Disease Research, Guangzhou, 510630 China

**Keywords:** 3D printing, Hydrogel scaffolds, Ferroptosis, Chemodynamic therapy, Tumor recurrence

## Abstract

Surgical resection to achieve tumor-free margins represents a difficult clinical scenario for patients with hepatocellular carcinoma. While post-surgical treatments such as chemotherapy and radiotherapy can decrease the risk of cancer recurrence and metastasis, growing concerns about the complications and side effects have promoted the development of implantable systems for locoregional treatment. Herein, 3D printed hydrogel scaffolds (designed as Gel-SA-CuO) were developed by incorporating one agent with multifunctional performance into implantable devices to simplify the fabrication process for efficiently inhibiting postoperative tumor recurrence. CuO nanoparticles can be effectively controlled and sustained released during the biodegradation of hydrogel scaffolds. Notably, the released CuO nanoparticles not only function as the reservoir for releasing Cu^2+^ to produce intracellular reactive oxygen species (ROS) but also serve as photothermal agent to generate heat. Remarkably, the heat generated by photothermal conversion of CuO nanoparticles further promotes the efficiency of Fenton-like reaction. Additionally, ferroptosis can be induced through Cu^2+^-mediated GSH depletion via the inactivation of GPX4. By implanting hydrogel scaffolds in the resection site, efficient inhibition of tumor recurrence after primary resection can be achieved in vivo. Therefore, this study may pave the way for the development of advanced multifunctional implantable platform for eliminating postoperative relapsable cancers.

**Graphical Abstract:**

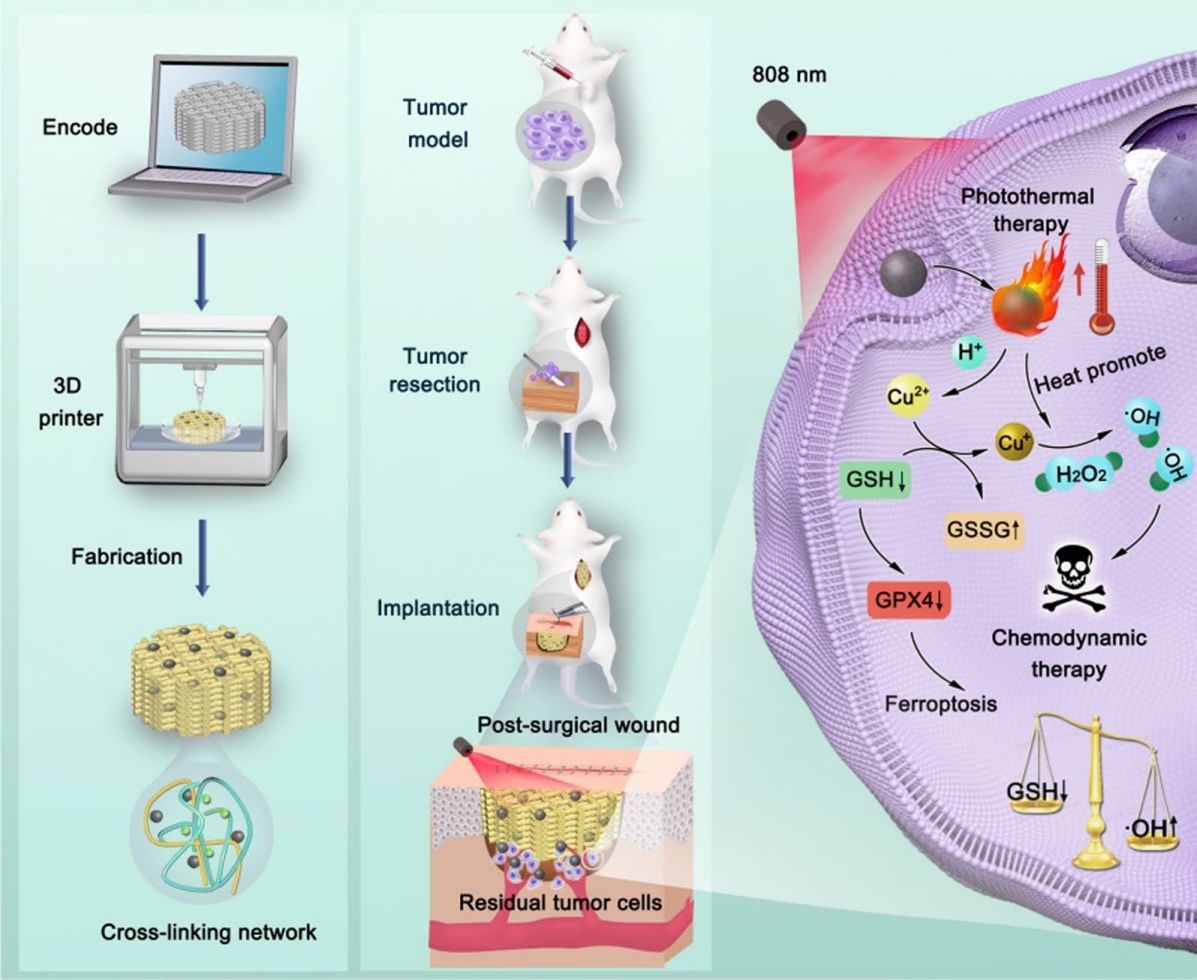

**Supplementary Information:**

The online version contains supplementary material available at 10.1186/s12951-022-01454-1.

## Introduction

As the most common histotype of liver cancer, hepatocellular carcinoma (HCC) accounts for 75–85% of primary liver malignancy, which contributes significantly to global morbidity and mortality [1, 2]. Although several treatments with proven survival benefits have been developed, surgical resection is still the first-line treatment in terms of overall survival and quality of life [3, 4]. However, it is technically precluded to achieve tumor-free margins especially for tumors with unique anatomy [5]. Ultimately, residual tumor cells increase the risk of cancer recurrence and metastasis. Additionally, post-surgical treatments such as chemotherapy and radiotherapy often induce complications and side effects [6]. Therefore, it is clinically impactful to explore adjuvant therapy to prevent inevitable local recurrence after surgical resection [7, 8].

Recently, advances in bioengineering technology have encouraged the development of biomaterial-based implants for the release of therapeutic agents in the surgical wound site, which has been considered as a promising strategy for inhibiting tumor recurrence [9, 10]. Compared to systemic administration, locally implants have shown great advantages including enhanced therapeutic dosage at the targeted site, decreased systemic toxicities, and adjustable release of payloads as well as the degradation of supporting matrix [11, 12]. To date, various implants such as wafers [13, 14], scaffolds [15–17], fibers [18–21], microneedle patches [22–25], hydrogels [26–31], and others [32–35] have been constructed for treating residual cancer after the resection surgery. In particular, 3D printed scaffolds have attracted considerable attention because of their striking advantages in customizing precisely matched shapes according to the anatomical structure of the patient’s defect with the aid of CT scanning and reconstruction software [36]. Currently, scaffolds loading two or more agents with synergistic therapy have been fabricated focusing on the combination of chemotherapy [37, 38], immunotherapy [39–44], photothermal therapy (PTT) [45–47], magnetothermal therapy [34, 48], chemodynamic therapy (CDT) [49–51], and so on [52]. However, the composition of scaffolds with synergistic therapy is more complicated than single modality, and complex functionalization processes are required [53, 54]. In addition, multiple synergistic systems limit the drug loading capacity of the scaffolds for every single therapeutic agent, which often devotes considerable efforts to adjust the ratios of different drugs to achieve the best therapeutic efficiency [55]. Furthermore, concerns on the biodegradation of the therapeutic agent as well as the scaffolds need to be solved. Thus, the development of biodegradable scaffolds loading a single agent with multiple effects is expected to be a straightforward and promising strategy to address the above issues for eliminating relapsable cancers.

Herein, we reported 3D printed hydrogel scaffolds (designed as Gel-SA-CuO) based on gelatin (Gel), sodium alginate (SA), and CuO nanoparticles for inhibiting tumor recurrence. The Gel is the product of collagen hydrolysate and SA is a kind of polysaccharide derived from brown seaweed, both of which have good biocompatibility and biodegradability [56]. The printable hydrogels were prepared by mixing CuO with Gel and SA in the presence of Ca^2+^, which was further used for fabricating hydrogel scaffolds with controlled size and shape by 3D printing, the prepared Gel-SA-CuO hydrogel scaffolds could fill in the resection defect and provide mechanical support for tissue. Moreover, Gel-SA-CuO hydrogel scaffolds provided controlled and sustained release of CuO nanoparticles during the biodegradation of hydrogel scaffolds. Besides, CuO nanoparticles with multifunctional properties endowed the hydrogel scaffolds with the synergistic anticancer ability for inhibiting tumor recurrence. On one hand, CuO nanoparticles could serve as photothermal agents for photothermal therapy. On the other hand, CuO could function as reservoir for releasing Cu^2+^ in acidic conditions to produce intracellular reactive oxygen species (ROS) via Fenton-like reaction. Remarkably, the heat generated by photothermal conversion of CuO further promoted the efficiency of Fenton-like reaction. Importantly, Gel-SA-CuO hydrogel scaffolds could induce ferroptosis through GSH consumption and inactivation of GPX4. Our study highlights the potential of incorporating one agent with multifunctional performance into implantable devices to simplify the fabrication process for inhibiting cancer recurrence.

## Experimental section

### Materials

Sodium alginate (SA), copper chloride dehydrate (CuCl_2_·2H_2_O), hydrogen peroxide (H_2_O_2_, 30 wt%), polyvinylpyrrolidone (PVP), sodium diethyldithiocarbamate trihydrate, and methylene blue were purchased from Aladdin Reagent Co., Ltd. Gelatin (Gel), calcium chloride anhydrous (CaCl_2_), thiazolyl blue (MTT) were obtained from Macklin Reagent Co., Ltd. 2′, 7′-Dichlorodihydrofluorescein diacetate (DCFH-DA) was purchased from Sigma Aldrich. Calcein AM was obtained from Yeasen Biotechnology Co., Ltd. Propidium iodide (PI) was bought from Cytiva (Shanghai, China). Annexin V-FITC/PI apoptosis detection kit was purchased from KeyGen Biotech Co., Ltd. The ThiolTracker Violet dye was obtained from ThermoFisher Scientific. GSH and GSSG assay kit and cellular glutathione peroxidase assay kit were bought from Beyotime Biotechnology. The GAPDH antibody was bought from Share-bio. The anti-glutathione peroxidase 4 (GP×4) antibody was purchased from Abcam. The HRP-conjugated AffiniPure goat anti-rabbit lgG(H + L) antibody was obtained from Proteintech. All the chemicals were used as received unless otherwise specified.

### Synthesis of CuO nanoparticles

CuO nanoparticles were prepared according to the following procedure. Firstly, 0.136 g CuCl_2_·2H_2_O was dissolved in 80 mL deionized water. Then 8.000 g PVP was added into the above solution and fully dissolved under the stirring by magnetic stirring apparatus. Next, 80 mL NaOH solution (0.03 M) and 1600 μL H_2_O_2_ (30 wt%) were successively added into the above mixture solution. After stirring for 40 min, the precipitates were collected by centrifuging at 6000 rpm for 5 min. After washing with water twice, these precipitates were transferred into an air oven at 60 °C for 24 h to get CuO nanoparticles, which were used for subsequent characterization and experiments.

### Preparation of Gel-SA-CuO hydrogels

Firstly, 0.5 g gelatin was dissolved in 5 mL deionized water at 50 °C. Then 0.012 g CaCl_2_ was added into gelatin solution with vigorous stirring. Next, 0.005 g CuO nanoparticles were dispersed in 3 mL deionized water by ultrasonication for 30 min, which was subsequently added into the mixture of gelatin and CaCl_2_ and stirred for 2 min. Finally, 0.42 g sodium alginate was added under stirring for 3 min to obtain Gel-SA-1CuO hydrogels. Similarly, the hydrogels containing 0 g, 0.008 g, 0.015 g, 0.025 g, 0.065 g, 0.1 g CuO nanoparticles were prepared by the same procedure, which were named as Gel-SA hydrogel, Gel-SA-2CuO hydrogel, Gel-SA-3CuO hydrogel, Gel-SA-4CuO hydrogel, Gel-SA-5CuO hydrogel, and Gel-SA-6CuO hydrogel.

### Fabrication of Gel-SA-CuO hydrogel scaffolds

Gel-SA-CuO hydrogel scaffolds were fabricated by a 3D printing machine (Changchun Ubbiotech Biotechnology Co., Ltd) through extruding Gel-SA-CuO hydrogels. Seven types of hydrogels (Gel-SA hydrogel, Gel-SA-1CuO hydrogel, Gel-SA-2CuO hydrogel, Gel-SA-3CuO hydrogel, Gel-SA-4CuO hydrogel, Gel-SA-5CuO hydrogel, and Gel-SA-6CuO hydrogel) were respectively loaded into the printing tube and then directly extruded from the tube head under compressed air with the pressure of 0.3–1.0 bar. The cube scaffolds were designed by solidwork 2014 software with side length of 13.0 mm and height of 2.3 mm (for photographing, photothermal assay, and swelling assay) and the print parameters were set as 10 mm/s of printing speed and 25% of filling rate by simplify3D software. Similarly, cylinder scaffolds with diameter of 8.5 mm and height of 2.5 mm (for degradation assay, release assay, ROS generation, and animal experiments) or diameter of 5.0 mm and height of 2.0 mm (for cell experiments) were designed and fabricated with the same procedure, except that the filling rate was respectively 35% and 40%. The printed hydrogel scaffolds were dried at room temperature for 48 h before use. These scaffolds were successively designated as Gel-SA, Gel-SA-1CuO, Gel-SA-2CuO, Gel-SA-3CuO, Gel-SA-4CuO, Gel-SA-5CuO, Gel-SA-6CuO.

### Materials characterization

The micro surface morphology and elemental mapping distribution of hydrogel scaffolds were detected by the scanning electron microscope equipped with energy dispersion spectrum (FEI, Quanta 400F). The particle diameter of CuO nanoparticles was evaluated by the nano-size analyzer (Anton Paar, Litesizer 500). The elemental binding energy of CuO nanoparticles and Gel-SA-CuO hydrogel scaffolds was tested by X-ray photoelectron spectroscopy (Thermo Fisher, ESCALab250). The phase composition of CuO nanoparticles was characterized by X-ray diffractometer (Panalytcal, Empyrean). Ultraviolet–visible absorption spectra were collected in a UV–vis spectrophotometer (Shimadzu, UV-2600).

### Rheological property of Gel-SA-CuO hydrogels

The rheological properties of hydrogels were evaluated by a rotational rheometer (Anton Paar GmbH, MCR302). Briefly, 1.0 mL hydrogels (Gel-SA hydrogel, Gel-SA-1CuO hydrogel, Gel-SA-2CuO hydrogel, Gel-SA-3CuO hydrogel, Gel-SA-4CuO hydrogel, Gel-SA-5CuO hydrogel, and Gel-SA-6CuO hydrogel) were injected on a platform via a 5 mL injection syringe, respectively. Then, the upper rounded plate with diameter of 25 mm was lowered to squeeze the hydrogel ink on the platform. Afterwards, the elastic modulus (G’) and loss modulus (G”) of hydrogels were measured and recorded in the condition of sequential shear strains from 0.1% to 150% at frequency of 1 Hz at 37 °C. Additionally, the shear viscosity of Gel-SA hydrogel, Gel-SA-1CuO hydrogel, Gel-SA-2CuO hydrogel, Gel-SA-3CuO hydrogel, Gel-SA-4CuO hydrogel, Gel-SA-5CuO hydrogel, and Gel-SA-6CuO hydrogel in the condition of varied shear rates from 0.1 to 1000 s^−1^ was measured and recorded at 37 °C.

### Swelling capacity of Gel-SA-CuO hydrogel scaffolds

The dry Gel-SA-CuO hydrogel scaffolds were firstly weighed and then put into wells in a 12-well cell plate (n = 3 for each group). Then, 2 mL deionized water was added. After incubation for 30 min, the remaining water in each well was removed by a pipette. Next, these wet scaffolds were gently taken out from wells and weighed. The swelling ratio of scaffolds was calculated by the following formula. The Sc = [(W_2_-W_1_)/W_1_] × 100%. The Sc represented the swelling capacity. W_2_ represented the weight of scaffolds under wet conditions, and W_1_ represented the weight of scaffolds under dry conditions.

### Degradation of the Gel-SA-CuO hydrogel scaffolds

The dry Gel-SA-CuO hydrogel scaffolds were weighed, respectively. Next, 1 mL sodium acetate-acetic acid buffer solution (pH = 6.5) was added. Then, these EP tubes containing scaffolds were shaken (37 °C, 100 rpm) for 3 h, 6 h, 12 h, and 24 h, respectively. At each time point, the hydrogel scaffolds were taken out and the solution was centrifuged and collected (n = 4 for each group). The residues of the hydrogel scaffolds were transferred into an oven at 50 °C. After completely drying, the residues of the hydrogel scaffolds were weighed. The degradation percentage was calculated using the following formula. The mass degradation percent W = [(W_a_-W_b_)/W_a_] × 100%. W_a_ represented the weight of scaffolds under dry conditions before degradation, and W_b_ represented the weight of scaffolds under dry conditions after degradation.

### Release of copper ions from Gel-SA-CuO hydrogel scaffolds

The standard curve of Cu^2+^ was firstly established as follows. 20 μL ammonia solution was added to 300 μL CuCl_2_·2H_2_O with various concentrations (5 μg/mL, 10 μg/mL, 25 μg/mL, 40 μg/mL, 50 μg/mL, 80 μg/mL) and the pH value was adjusted to be around 8. Then, 200 μL sodium diethyldithiocarbamate trihydrate (0.1 g/mL) was added to induce the chromogenic reaction. The absorption values at 452 nm were recorded to get the standard curve by a microplate reader (BioTek, Synergy H1).

The released Cu^2+^ from Gel-SA-CuO hydrogel scaffolds were evaluated as follows. Gel-SA-CuO hydrogel scaffolds were incubated with 1 mL sodium acetate-acetic acid buffer solution (pH = 6.5) for the indicated time. Then, we collected 100 μL from the above solution and added another 200 μL sodium acetate-acetic acid buffer solution. The above 300 μL solutions were mixed with 20 μL ammonia solution in a 48-well culture plate. Afterwards, 200 μL solution of sodium diethyldithiocarbamate trihydrate (0.1 g/mL) was added. Finally, the absorption values at 452 nm were recorded by a microplate reader. Quantitative analysis of Cu^2+^ was performed according to the standard curve of Cu^2+^ ions. The percentage of released Cu^2+^ from Gel-SA-CuO hydrogel scaffolds was also calculated by dividing the weight of released Cu^2+^ into total weight of Cu in the Gel-SA-CuO hydrogel scaffolds.

### Photothermal behavior of Gel-SA-CuO hydrogel scaffolds

Gel-SA-CuO hydrogel scaffolds were respectively put into wells in a 12-well plate (one scaffold for each well, n = 4 for each group). Then, the NIR laser (LASEVER, LSR808H-4 W-FC) was utilized to irradiate Gel-SA-CuO hydrogel scaffolds at 0.6 W/cm^2^. In the meantime, the real-time temperature was monitored by a thermal imager (Dongguan Xintai Instrument Co., Ltd, Hti HT-19). To study the influence of power density on the photothermal behavior of Gel-SA-CuO hydrogel scaffolds, NIR laser with different power densities (0.5 W/cm^2^, 0.6 W/cm^2^, 0.7 W/cm^2^, 0.8 W/cm^2^, and 0.9 W/cm^2^) was used to irradiate Gel-SA-4CuO hydrogel scaffold, and temperature changes were recorded (n = 4). To investigate the photothermal performance of Gel-SA-CuO hydrogel scaffolds under wet conditions, 2 mL deionized water was added into each scaffold for 30 min, and then the remaining water was removed. Gel-SA-CuO hydrogel scaffolds under wet conditions were irradiated by NIR laser at 1.3 W/cm^2^, and the temperature was recorded. Similarly, the NIR laser with power densities of 0.9 W/cm^2^, 1.0 W/cm^2^, 1.1 W/cm^2^, 1.2 W/cm^2^, 1.3 W/cm^2^ was employed to irradiate wet Gel-SA-6CuO hydrogel scaffold to study the effect of power density on the photothermal behavior of Gel-SA-6CuO hydrogel scaffold under wet conditions.

### The ROS generation capability of Gel-SA-CuO hydrogel scaffolds

The hydrogel scaffolds (Gel-SA, Gel-SA-1CuO, Gel-SA-2CuO, Gel-SA-3CuO, Gel-SA-4CuO, Gel-SA-5CuO, and Gel-SA-6CuO hydrogel scaffolds) were mixed with 1 mL sodium acetate-acetic acid buffer (pH 6.5). After shaking at 37 °C with a speed of 100 rpm for 24 h, 700 μL solutions from each group were collected and put into wells in a 48-well plate (n = 3 for each group). The ROS generation capability of Gel-SA-CuO hydrogel scaffolds was evaluated by the methylene blue degradation assay as follows. Firstly, 10 μL methylene blue (1 mg/mL) was added to the well and mixed homogeneously by pipette. Next, 100 μL H_2_O_2_ (1 M) and 100 μL glutathione (GSH, 10 mmol/L) were respectively added into each well and stirred homogeneously. Then the plate was transferred to an oven with a set temperature of 37 °C for 180 min. Finally, the absorption spectra of the solution in each well of the plate were recorded by a UV–Vis spectrophotometer.

In order to investigate the effect of degradation time on the ROS generation capability, Gel-SA-6CuO hydrogel scaffold was immersed into 1 mL sodium acetate-acetic acid buffer and then shaken at 37 °C with the speed of 100 rpm for 3 h, 6 h, 12 h, and 24 h, respectively. At each time point, 700 μL solutions were collected for evaluating the ROS generation capability by the methylene blue degradation assay as described above.

To study the influence of reaction temperature on ROS generation capability, Gel-SA-6CuO scaffold was immersed in 1 mL buffer solution and shaken for 24 h. Then, 700 μL solutions were collected, followed by adding 10 μL methylene blue, 100 μL H_2_O_2_, and 100 μL GSH in sequence. After reacting for 90 min at indicated temperature (25 °C, 37 °C, 45 °C, and 53 °C), the absorption spectra of the solution in each well of the plate were detected by a UV–Vis spectrophotometer.

To study the influence of NIR irradiation on ROS generation capability, Gel-SA-6CuO scaffold was mixed with 250 μL sodium acetate-acetic acid buffer and shaken for 0.5 h. Then, NIR laser with power densities of 1.3 W/cm^2^, 1.4 W/cm^2^, 1.5 W/cm^2^ was employed to irradiate scaffolds for 10 min. After irradiating, 750 μL sodium acetate-acetic acid buffer was added and shaken at 37 °C for additional 23.5 h. Afterwards, 700 μL solutions were collected for evaluating the ROS generation capability by degradation of methylene blue assay. After reacting for 180 min, the absorption spectra were detected by a UV-Vis spectrophotometer.

### In vitro cytotoxicity assay

All the hydrogel scaffolds were sterilized under ultraviolet light for 1 h before the cell experiment. Murine hepatocellular carcinoma H22 cells were used for in vitro investigation. The cell culture medium was composed of 89% RPMI medium 1640 basic (1×), 10% fetal bovine serum (FBS), and 1% penicillin/streptomycin. Briefly, different kinds of hydrogel scaffolds (Gel-SA, Gel-SA-1CuO, Gel-SA-2CuO, Gel-SA-3CuO, Gel-SA-4CuO, Gel-SA-5CuO, and Gel-SA-6CuO hydrogel scaffolds) were put into wells in a 48-well plate (n = 4), followed by adding 1200 μL cell culture medium containing 1.5 × 10^5^ H22 cells in each well. Cells cultured without hydrogel scaffolds were set as control group. The cell incubation condition in this study was set as 37 °C, 5% CO_2_, and saturated humidity. After incubation for 24 h, the medium containing H22 cells from each group was transferred into an Eppendorf tube and then centrifuged. After removing the supernatant, 500 μL fresh culture medium was added, and cell pellets were dispersed by gently pipetting. Then, 50 μL MTT (5 mg/mL in PBS) was added. After 4 h, each Eppendorf (EP) tube was centrifuged to precipitate formazan, and the supernatant was removed gently. Next, 400 μL DMSO was added into each Eppendorf tube to dissolve purple formazan. Finally, the solution was transferred to wells in a 48-well plate, and the absorption value at 570 nm was recorded by a microplate reader (Synergy H1, BioTek). In order to investigate the effect of culture time on the viability, Gel-SA hydrogel scaffolds and Gel-SA-6CuO hydrogel scaffolds were incubated with 1.5 × 10^5^ H22 cells for 3 h, 6 h, 12 h, and 24 h, respectively. At each time point, H22 cells were collected, and the MTT assay was conducted.

In order to investigate the combined effect of photothermal and chemodynamic therapies on cell viability, seven types of scaffolds (Gel-SA, Gel-SA-1CuO, Gel-SA-2CuO, Gel-SA-3CuO, Gel-SA-4CuO, Gel-SA-5CuO, and Gel-SA-6CuO hydrogel scaffolds) were added into wells in a 48-well plate (n = 4 for each group). Then, 200 μL cell culture medium containing 1.5 × 10^5^ H22 cells was added into each well, and the NIR laser at 1.5 W/cm^2^ was employed to irradiate scaffolds for 10 min. Then 1000 μL fresh culture medium was added, and the incubation was performed for another 24 h. Finally, cells were collected, and the MTT assay was conducted.

### Live/dead staining assay

To further examine the cell viability of H22 cells, the live/dead staining was conducted with calcein AM and propidium iodide (PI). Gel-SA hydrogel scaffolds and Gel-SA-4CuO hydrogel scaffolds were added into wells in a 48-well plate, respectively. Then, 1200 μL cell culture medium containing 1.5 × 10^5^ H22 cells was added into each well. After culture for 24 h, H22 cells in each group were collected and transferred into 1.5 mL Eppendorf tubes. After centrifuging and removing the supernatant, cell pellets were dispersed in 500 μL PBS. Then, the live/dead staining assay was performed as follows. Briefly, 50 μL PBS solution containing calcein AM (5 μM) and PI (7.5 μM) were added into the above cells and then incubated at 37 °C for 15 min. Cells were centrifuged and washed with PBS twice. Finally, cells were observed and photographed by the inverted fluorescence microscope (Axio Observer 3, Zeiss). For Gel-SA + NIR and Gel-SA-4CuO + NIR group, 200 μL cell culture medium containing 1.5 × 10^5^ H22 cells was added, and then the NIR laser at 1.5 W/cm^2^ was used to irradiate Gel-SA and Gel-SA-4CuO hydrogel scaffolds for 10 min. Afterwards, 1000 μL culture medium was added and incubation was performed for another 24 h. After centrifuging and removing the supernatant, cell pellets were dispersed in 500 μL PBS. Then, the live/dead staining assay was performed as described above.

### Intracellular detection of ROS

Gel-SA, Gel-SA-4CuO hydrogel scaffolds were added into wells in a 48-well plate, respectively. Then, 200 μL cell culture medium containing 1.5 × 10^5^ H22 cells were added into each well. For groups of Gel-SA + NIR and Gel-SA-4CuO + NIR, the NIR laser at 1.5 W/cm^2^ was employed to irradiate scaffolds for 10 min. Subsequently, 1000 μL cell culture medium was added into each well for all groups. After culturing for 5 h, cells were gently transferred into 1.5 mL EP tube and centrifuged to remove the supernatant. Then 500 μL PBS containing 5 μM DCFH-DA was added into each EP tube and cultured for 20 min. Cells were centrifuged and washed with 500 μL PBS solution. Finally, cells were observed and photographed by the inverted fluorescence microscope (Axio Observer 3, Zeiss). The parameter of the microscope for all the samples were kept consistent including light source power (120 W), exposing time (50 ms), brightness/contrast, excitation wavelength (470/40 nm), and emission wavelength (525/50 nm).

### Measurement of intracellular GSH/GSSG ratio

Intracellular GSH/GSSG ratio was measured using a GSH and GSSG assay Kit (Beyotime Biotechnology). Briefly, 500 μL cell culture medium containing 3.0 × 10^5^ H22 cells was added into each well containing Gel-SA hydrogel scaffolds and Gel-SA-6CuO hydrogel scaffolds of a 48-well plate. For Gel-SA + NIR group and Gel-SA-6CuO + NIR group, Gel-SA and Gel-SA-6CuO scaffolds were irradiated by NIR laser with 1.5 W/cm^2^ for 10 min. Subsequently, 700 μL of fresh medium was added in each well for all groups. After incubation for 6 h, cells were collected and intracellular GSH/GSSG ratio was detected according to the instruction of GSH and GSSG assay Kit.

### Measurement of GPX4 protein expression and GPx activity

Firstly, 500 μL cell culture medium containing 3.0 × 10^5^ H22 cells was added into each well containing Gel-SA and Gel-SA-6CuO hydrogel scaffolds. For Gel-SA + NIR and Gel-SA-6CuO + NIR groups, Gel-SA and Gel-SA-6CuO hydrogel scaffolds were irradiated by NIR laser with 1.5 W/cm^2^ for 10 min, followed by adding 700 μL fresh cell culture medium into each well. After incubation for 6 h, cells were collected and lysed by cell lysis buffer for Western and IP (Beyotime Biotechnology). Then, the proteins were quantified by BCA Protein Assay Kit (Beyotime Biotechnology). Next, Protein samples from each group were separated by 12% GenScript SurePAGE Bis–Tris gels (GenScript) and then transferred to a PVDF membrane (Merck Millipore). Subsequently, the membranes were blocked in TBST containing 5% bovine serum albumin (BSA) and then incubated with primary antibodies (GPX4, Abcam; GAPDH, *S*hare-bio). After washing, membranes were incubated with horseradish peroxidase-conjugated secondary antibody (goat anti-rabbit, Proteintech) and electrochemiluminescence substrate (Solarbio) before visualization using a chemiluminescence blotting detection system (Syngene G: BOX Chemi XX6, Gene Company Limited). Additionally, the activity of intracellular GPx was measured by the Cellular Glutathione Peroxidase Assay Kit (Beyotime Biotechnology) according to the manufacturer’s protocol.

### Detection of cell apoptosis

For Gel-SA hydrogel scaffolds group and Gel-SA-6CuO hydrogel scaffolds group, 1.5 × 10^5^ H22 cells suspended in 1200 μL culture medium were added into each well, followed by adding Gel-SA and Gel-SA-6CuO scaffolds into wells in a 48-well plate. The culture temperature was 37 ℃ and the density of CO_2_ was 5%. After culturing for 24 h, the culture medium containing H22 cells was transferred into 1.5 mL EP tube. After centrifuging and removing the supernatant, cell pellets were washed with 500 μL PBS solution. Then, 500 μL binding buffer was added in cell pellets in EP tube, followed by the addition of 5 μL Annexin V-FITC and 5 μL propidium iodide (PI). After incubation for another 15 min in an incubator, cells were analyzed by a flow cytometric machine (BD LSRFortessa). For Gel-SA + NIR and Gel-SA-6CuO scaffolds + NIR group, 200 μL culture medium containing 1.5 × 10^5^ H22 cells was added into each well, and NIR laser at 1.5 W/cm^2^ was employed to irradiate scaffolds for 10 min. Then, 1000 μL cell culture medium was added into each well, and incubation was performed for another 24 h. After being stained with Annexin V-FITC and PI, cells were analyzed by the flow cytometric machine.

### In vivo investigation of Gel-SA-CuO hydrogel scaffolds

All animal procedures were performed in accordance with the Guidelines for Care and Use of Laboratory Animals of Sun Yat-sen University, and approved by the Animal Ethics Committee of Sun Yat-sen University. Female balb/c mice at the age of 4–6 weeks were used to establish surgical resection models. Briefly, 300 μL PBS solution containing 8 × 10^6^ H22 cells was injected into the abdominal cavity of a female balb/c mouse (n = 5). On day 7, the H22 cells in the abdominal cavity were collected. Then, cells were centrifuged at 2000 rpm for 5 min. After washing with PBS solution thrice, 8.0 × 10^6^ cells in 100 μL PBS were injected into the balb/c mouse subcutaneously to construct the tumor model. After 9 days, the tumor volume reached approximately 750 mm^3^, and the mice were randomly divided into five groups (control, Gel-SA, Gel-SA + NIR, Gel-SA-6CuO, and Gel-SA-6CuO + NIR; n = 4 for each group). Then, the tumor in each group was resected about 70% in anesthetized conditions. Subsequently, Gel-SA hydrogel scaffolds and Gel-SA-6CuO hydrogel scaffolds were implanted into the surgical defects, respectively. The mice without implanting scaffolds were set as the control group. The day when the scaffolds were implanted was marked as day 0 for recording data. The tumor volume and mice weight were measured every two days. For Gel-SA + NIR and Gel-SA-6CuO + NIR group, the mice were irradiated by NIR laser for 10 min once a day for successive four days. The tumor volume was calculated as follows: tumor volume (V) = (tumor length) × (tumor width)^2^/2. On day 10, the mice were executed, and the tumors from each group were harvested, weighed, and photographed. Tumor tissues and main organs including liver, heart, lung, spleen, and kidney were collected, immersed into 4% paraformaldehyde overnight, and embedded in paraffin for hematoxylin and eosin (H&E) staining. To investigate the ROS generation, tumors from different groups were stained with the DCFH-DA (30 uM) for 40 min and DAPI (1 μg/mL) for 10 min. Besides, tumors were stained with the ThiolTracker Violet dye (30 uM) for 40 min and DAPI (1 μg/mL) for 10 min to investigate the cellular level of reduced GSH. Additionally, Anti-Ki67 Rabbit pAb (Servicebio) was used to monitor the cell proliferation of tumor cells according to the manufacturer’s protocol.

### Statistical analysis

Statistical analysis was performed using GraphPad Prism 7.0. Statistical analysis was performed using one-way analysis of variance (ANOVA) followed by Tukey’s post hoc tests. The statistical significance was defined as *p < 0.05, **p < 0.01, ***p < 0.001.

## Results and discussion

### Fabrication and characterization of Gel-SA-CuO hydrogel scaffolds

CuO nanoparticles were initially synthesized by the reaction of CuCl_2_, H_2_O_2_, and NaOH using polyvinylpyrrolidone (PVP) as the stabilizer. The microscopic morphology of CuO was characterized by scanning electron microscopy (SEM). As shown in Fig. 2a, the prepared CuO exhibited a sphere-like shape. The hydrodynamic diameter of as-prepared CuO was about 500 nm based on the dynamic light scattering analysis, further confirming the successful synthesis of CuO nanoparticles (Fig. 2b). Additionally, X-ray diffraction (XRD) pattern of synthesized CuO nanoparticles was in good agreement with the JCPDS card of CuO (JCPSD 89–2529), which indicated the monoclinic phase of CuO (Fig. 2c).

**Fig. 1:**
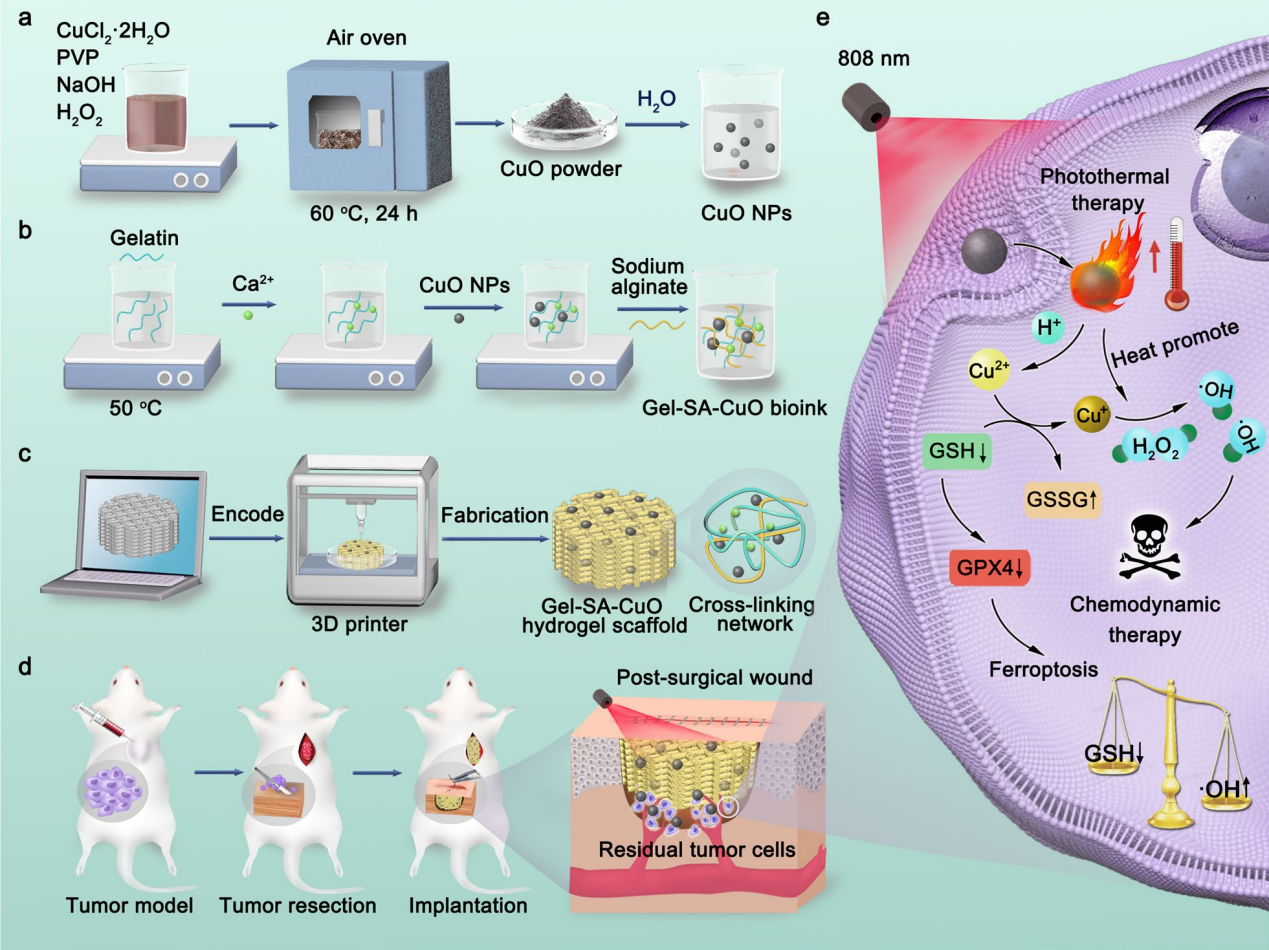
Schematic illustration of 3D printed Gel-SA-CuO hydrogel scaffolds for inhibiting postoperative tumor recurrence. **a** CuO nanoparticles were initially synthesized by the reaction of CuCl_2_, H_2_O_2_, and NaOH using polyvinylpyrrolidone (PVP) as the stabilizer. **b** By mixing CuO nanoparticles with gelatin and sodium alginate, Gel-SA-CuO hydrogels were successfully prepared. **c** Gel-SA-CuO hydrogels were used as bioink for fabricating 3D printed Gel-SA-CuO hydrogel scaffolds. **d** Through implanting Gel-SA-CuO hydrogel scaffolds in the resection site, efficient inhibition of tumor recurrence after primary resection can be achieved. **e** The anticancer mechanism was associated with glutathione depletion induced ferroptosis and photothermia augmented chemodynamic therapy

**Fig. 2:**
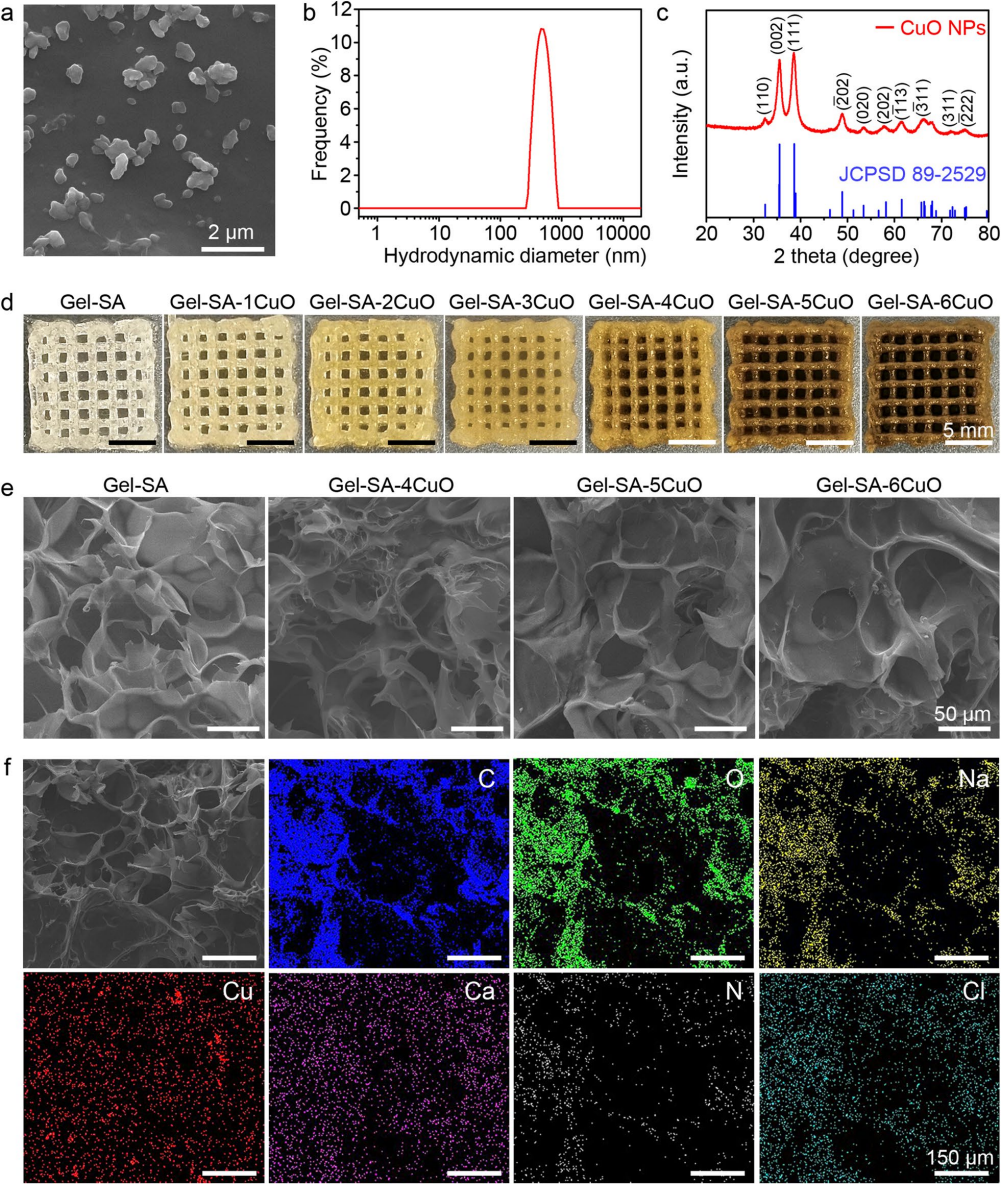
Characterization of prepared CuO nanoparticles and Gel-SA-CuO hydrogel scaffolds. **a** SEM image of CuO nanoparticles. **b** Hydrodynamic diameter of CuO nanoparticles. **c** XRD pattern of CuO nanoparticles. The vertical lines indicate the position and relative intensity of diffraction peaks in JCPSD 89–2529 card file. **d** Digital photographs of Gel-SA scaffold, Gel-SA-1CuO scaffold, Gel-SA-2CuO scaffold, Gel-SA-3CuO scaffold, Gel-SA-4CuO scaffold, Gel-SA-5CuO scaffold, and Gel-SA-6CuO scaffold under wet conditions from left to right. **e** SEM images of Gel-SA scaffold, Gel-SA-4CuO scaffold, Gel-SA-5CuO scaffold, and Gel-SA-6CuO scaffold from left to right. **f** Element mapping of C, O, Na, Cu, Ca, N, and Cl for Gel-SA-6CuO scaffold

By mixing CuO nanoparticles with Gel and SA, the Gel-SA-CuO hydrogels were successfully prepared as inks for fabricating 3D printed Gel-SA-CuO hydrogel scaffolds. Figure 1d shows the photos of seven kinds of hydrogel scaffolds under wet conditions. In contrast to the white color of Gel-SA hydrogel scaffold, the color of Gel-SA-CuO hydrogel scaffolds changed from light yellow to dark yellow with the increase of CuO nanoparticles content (Additional file 1: Fig. S1). After drying, the hydrogel scaffolds showed little change regarding the structure (Additional file 1: Fig. S2). Scanning electron microscope (SEM) images revealed that lots of macropore structures were inside all the scaffolds and interconnected (Fig. 2e). Notably, the incorporation of CuO nanoparticles did not change the inner structure of hydrogel scaffolds. Elemental mapping clearly showed the distribution of C, N, O, Cu, Ca, Na, Cl elements on the surface of the fabricated hydrogel scaffolds (Fig. 2f). Especially, the distribution of Cu element was relatively homogeneously, which indicated that CuO nanoparticles were successfully evenly incorporated in the hydrogel scaffolds.

**Fig. 3:**
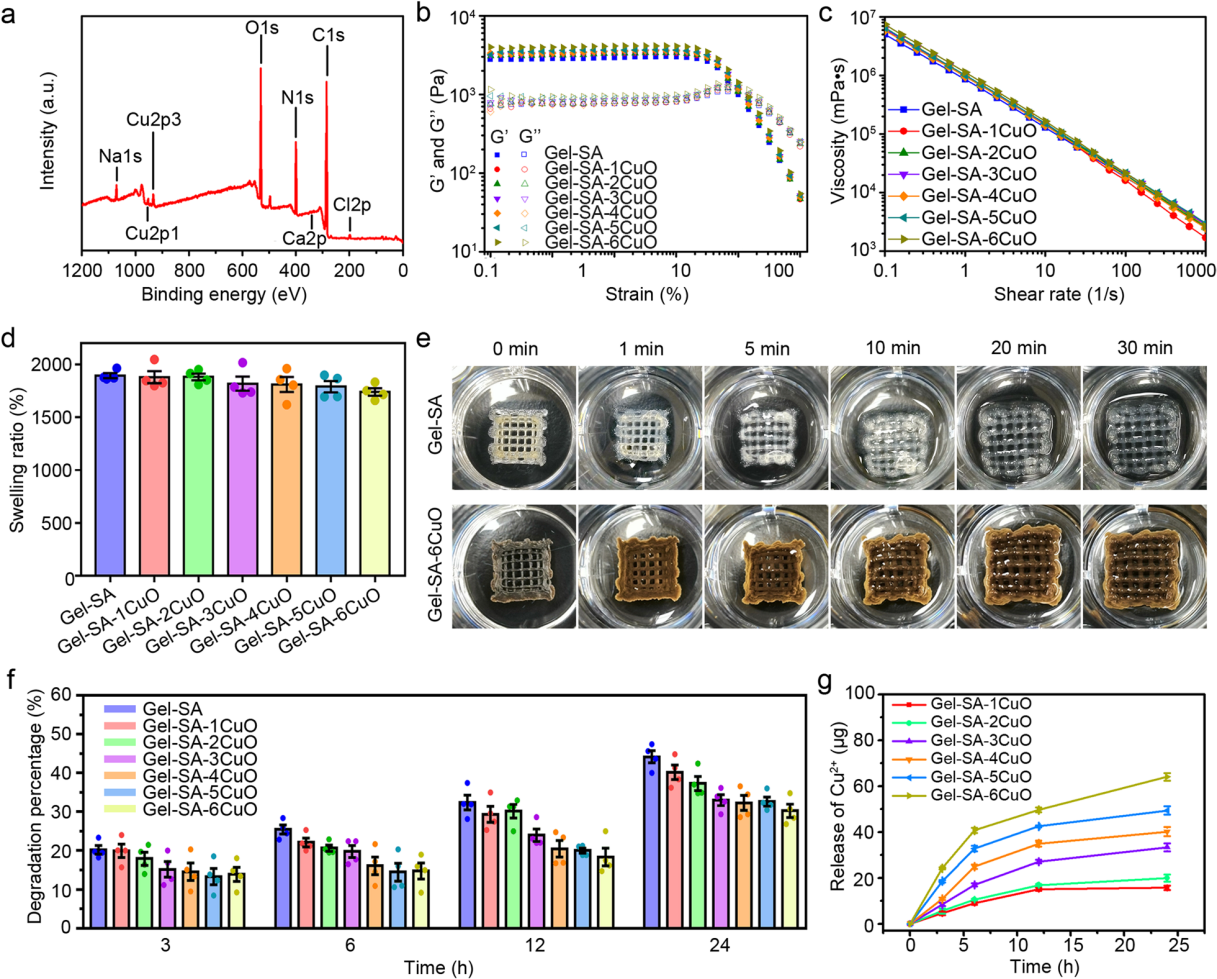
Composition, rheological performance and release behavior of Gel-SA-CuO hydrogel scaffolds. **a** XPS spectra of Gel-SA-6CuO scaffold. **b** Strain amplitude sweep measurements of Gel-SA hydrogel, Gel-SA-1CuO hydrogel, Gel-SA-2CuO hydrogel, Gel-SA-3CuO hydrogel, Gel-SA-4CuO hydrogel, Gel-SA-5CuO hydrogel, and Gel-SA-6CuO hydrogel at a fixed frequency of 1 Hz. **c** Shear thinning behaviors of Gel-SA hydrogel, Gel-SA-1CuO hydrogel, Gel-SA-2CuO hydrogel, Gel-SA-3CuO hydrogel, Gel-SA-4CuO hydrogel, Gel-SA-5CuO hydrogel, and Gel-SA-6CuO hydrogel. **d** The swelling ratio of Gel-SA-CuO scaffolds. **e** Representative photographs of swelling behavior of Gel-SA and Gel-SA-6CuO scaffolds. **f** The degradation property of Gel-SA-CuO scaffolds. **g** Time-dependent release of Cu^2+^ from Gel-SA-CuO scaffolds

The successful loading of CuO nanoparticles into the scaffolds was further determined by X-ray photoelectron spectroscopy (XPS), which revealed the presence of copper, calcium, oxygen, nitrogen, and chlorine (Fig. 3a). In particular, two satellite peaks in Cu 2p XPS spectrum demonstrated the bivalent oxidation state of the elemental Cu (Additional file 1: Fig. S3). Considering that the hydrogel scaffolds need to have sufficient mechanical strength after implantation in the surgical defect, the rheological property of the hydrogels were investigated by measuring the storage modulus (G′) and loss modulus (G″). It has been reported that the hydrogels present solid state and mainly occur elastic deformation when G′ > G″, and present liquid state and mainly occur viscous deformation when G’ < G″ [57, 58]. As shown in Fig. 3b, the G′ and G″ of seven types of hydrogels remained stable at low shear strain and the G′ was higher than G″, which indicated that they have good elasticity at low shear strain and paved the way for printability. With the increase of shear strain, the G′ and G″ gradually intersected in one point and then separated gradually. Additionally, the viscosity of seven types of hydrogels decreased with the increase of shear rate, indicating its shear-thinning property is favorable for printability (Fig. 3c). Besides, the swelling ratios of Gel-SA, Gel-SA-1CuO, Gel-SA-2CuO, Gel-SA-3CuO, Gel-SA-4CuO, Gel-SA-5CuO, and Gel-SA-6CuO hydrogel scaffolds were 1894.79%, 1880.01%, 1882.07%, 1818.45%, 1810.27%, 1789.57%, and 1740.06%, respectively, which indicated that the swelling capacity of the hydrogel scaffolds presented slight decrease with the increase of CuO nanoparticles (Fig. 3d). Especially, it was visibly obvious that the swelling capacity of Gel-SA-6CuO hydrogel scaffolds decreased when compared with Gel-SA hydrogel scaffolds (Fig. 3e and Additional file 1: Figs. S4 and S5). Furthermore, it was observed that degradation percentages of all the hydrogel scaffolds gradually enhanced with the increase of time (Fig. 3f and Additional file 1: Fig. S6). Additionally, more amount of Cu^2+^ was observed to be released from the Gel-SA-CuO hydrogel scaffolds with the increase of time (Fig. 3g). At the same time, the amount of released Cu^2+^ gradually increased from Gel-SA-1CuO scaffold to Gel-SA-6CuO scaffold, which was ascribed to the increased amount of Cu in the hydrogel scaffolds. The calculated percentage of released Cu^2+^ from hydrogel scaffolds decreased gradually from Gel-SA-1CuO scaffold to Gel-SA-6CuO scaffold at the same time point (Additional file 1: Fig. S7). All the above results substantiate Gel-SA-CuO hydrogel scaffolds own the proper degradation rate and mechanical properties as well as the sustained-release profiles.

### Photothermal property of Gel-SA-CuO hydrogel scaffolds

**Fig. 4:**
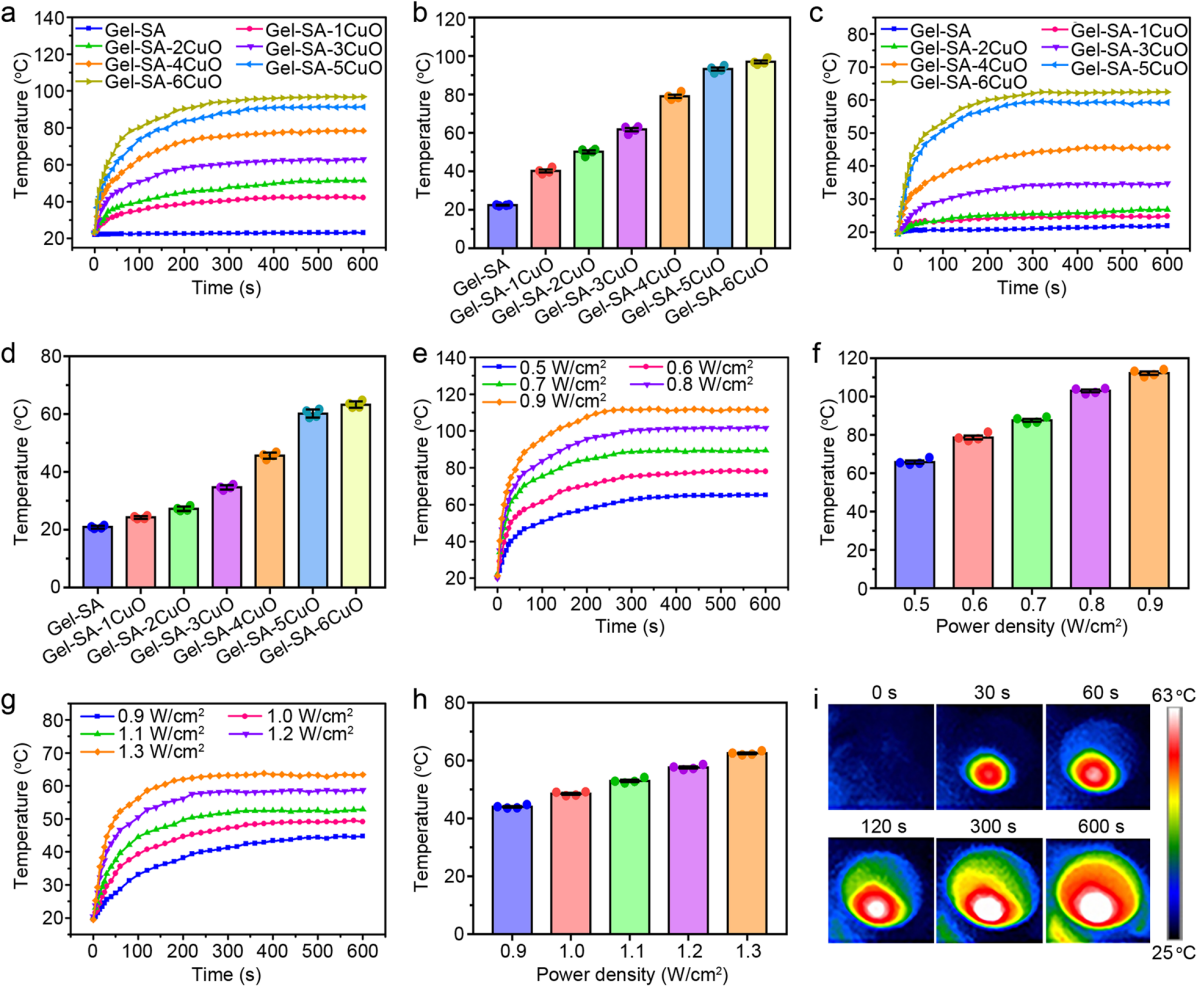
Photothermal performance of Gel-SA-CuO hydrogel scaffolds. Temperature changing curves (**a**) and the average equilibrium temperatures (**b**) of Gel-SA-CuO scaffolds exposed to NIR laser at 0.6 W/cm^2^ under dry conditions. Temperature changing curves (**c**) and the average equilibrium temperatures (**d**) of Gel-SA-CuO scaffolds exposed to NIR laser at 1.3 W/cm^2^ under wet conditions. Temperature changing curves (**e**) and the average equilibrium temperatures (**f**) of Gel-SA-4CuO scaffolds under dry conditions exposed to different power density of NIR laser. Temperature changing curves (**g**) and the average equilibrium temperatures (**h**) of Gel-SA-6CuO scaffolds under wet conditions exposed to different power density of NIR laser. **i** The representative photothermal images of Gel-SA-6CuO scaffolds exposed to NIR laser at 1.3 W/cm^2^ at indicated time points under wet conditions

The photothermal conversion of Gel-SA-CuO hydrogel scaffolds was investigated by recording the temperature of the scaffolds with near-infrared (NIR) laser irradiation. When exposed to NIR laser, Gel-SA hydrogel scaffold under dry conditions showed no temperature change (Fig. 4a and b). In contrast, the equilibrium temperatures of hydrogel scaffolds from Gel-SA-1CuO to Gel-SA-6CuO were gradually increased to 40.20 °C, 50.13 °C, 61.70 °C, 79.10 °C, 93.23 °C, and 97.00 °C under the same irradiation condition. We also found the equilibrium temperatures of hydrogel scaffolds from Gel-SA-1CuO to Gel-SA-6CuO under wet conditions were 24.25 °C, 27.25 °C, 34.65 °C, 45.68 °C, 60.23 °C, and 63.33 °C with NIR laser irradiation at 1.3 W/cm^2^ (Fig. 4c and d). These results indicated the environmental humidity exhibited significant influence on the photothermal property of Gel-SA-CuO hydrogel scaffolds. The equilibrium temperatures in wet condition were lower than those in dry condition may be attributed to the following reasons. Firstly, the PBS solution could absorb a certain amount of NIR laser when NIR laser went through PBS solution. Secondly, PBS solution could absorb some heat energy released from the hydrogel scaffolds when exposed to NIR laser. Thirdly, the PBS solution could lower the thermal conductivity of hydrogel scaffolds [37]. Additionally, the effect of power density of NIR laser on the photothermal behavior of Gel-SA-CuO hydrogel scaffolds was also studied. It was demonstrated that the equilibrium temperature of Gel-SA-4CuO hydrogel scaffold gradually increased from 65.95 °C to 112.35 °C under dry conditions, when power density of NIR laser increased from 0.5 W/cm^2^ to 0.9 W/cm^2^ (Fig. 4e and f). Correspondingly, the equilibrium temperature of Gel-SA-6CuO hydrogel scaffold increased from 44.05 °C to 62.55 °C under wet conditions, when the power density of NIR laser increased from 0.9 W/cm^2^ to 1.3 W/cm^2^ (Fig. 4g and h). The representative photothermal images of Gel-SA-6CuO scaffold exposed to NIR laser with the power density of 1.3 W/cm^2^ further verified the good photothermal conversion effect (Fig. 4i). Together, all the above results suggest CuO nanoparticles endowed hydrogel scaffolds with good photothermal property, which could potentially function in killing cancer cells and even combating multi-drug-resistant tumors [59].

### Chemodynamic behavior of Gel-SA-CuO hydrogel scaffolds

**Fig. 5:**
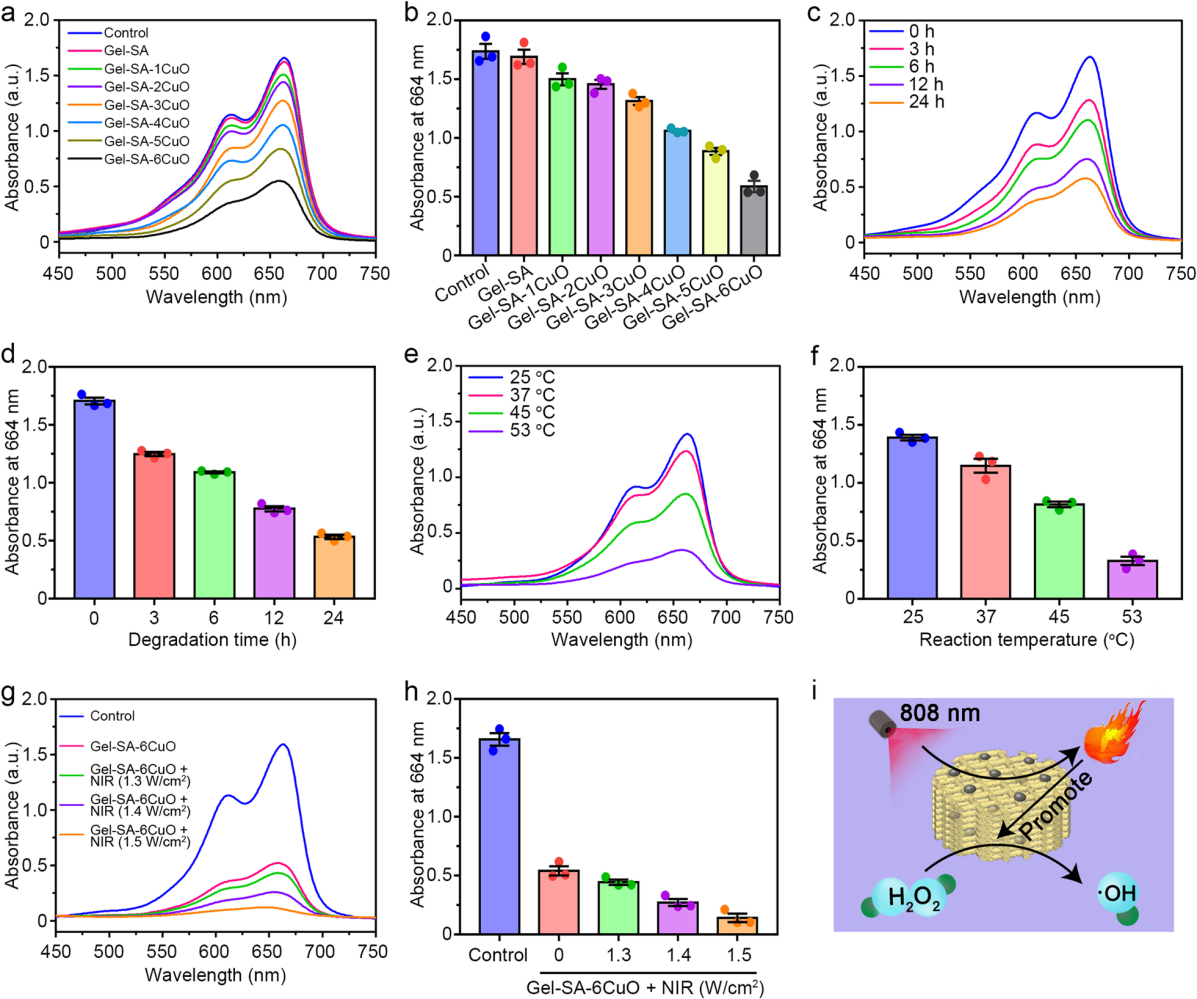
Chemodynamic behavior of Gel-SA-CuO hydrogel scaffolds. The absorbance curves (**a**) and absorption values at 664 nm (**b**) of methylene blue mixed with released solution from indicated scaffolds. The influence of reaction time (**c** and **d**), reaction temperature (**e** and **f**), and power density (**g** and **h**) on the degradation of methylene blue. **i** Schematic illustration of enhanced catalytic activity of Gel-SA-CuO by photothermal effect induced temperature increase

Previous studies have reported that Cu^2+^ could be reduced by intracellular GSH to Cu^+^, which further reacted with H_2_O_2_ to generate ·OH through Fenton-like reaction [60–62]. To validate the chemodynamic performance of Gel-SA-CuO hydrogel scaffolds, we mixed the released solutions from scaffolds with GSH and H_2_O_2_ to evaluate the degradation of methylene blue. As shown in Fig. 5a, it was found that the absorption spectrum of Gel-SA was similar to the control group, which suggested that scaffold without CuO nanoparticles did not show chemodynamic performance. In contrast, the absorption values at 664 nm for Gel-SA-CuO hydrogel scaffold were lower than that of the control group, which could be explained by the generation of ·OH induced by reaction of Cu^2+^ ions, glutathione, and H_2_O_2_ to make methylene blue fade [63–66]. Besides, with the increased content of CuO in the hydrogel scaffolds, a larger amount of ·OH could be produced (Fig. 5b). With the extension of degradation time of the scaffolds, the absorption value of methylene blue at 664 nm gradually decreased (Fig. 5c and d). The plausible explanation was that more Cu^2+^ ions were released with the increase of degradation time, thus leading to the generation of more ·OH. Moreover, increased reaction temperature also decreased the absorption value of methylene blue at 664 nm (Fig. 5e and f). This phenomenon may be ascribed to the fact that high temperatures could accelerate the Fenton-like reaction [53, 67–70]. Furthermore, we conducted the NIR laser irradiation on the Gel-SA-6CuO hydrogel scaffold during the process of methylene blue degradation (Fig. 5g and h). Intriguingly, it was observed that the NIR laser irradiation contributed to the decrease of the absorption value of methylene blue at 664 nm. As the temperature has a significant influence on the catalytic activity of Fenton agents, the heat generated from the photothermal conversion could improve the efficiency of producing ·OH (Fig. 5i). Taken together, Gel-SA-CuO hydrogel scaffolds have great potential as an effective PTT-enhanced CDT reagent for cancer therapy.

### In vitro anticancer efficacy of Gel-SA-CuO hydrogel scaffolds

**Fig. 6:**
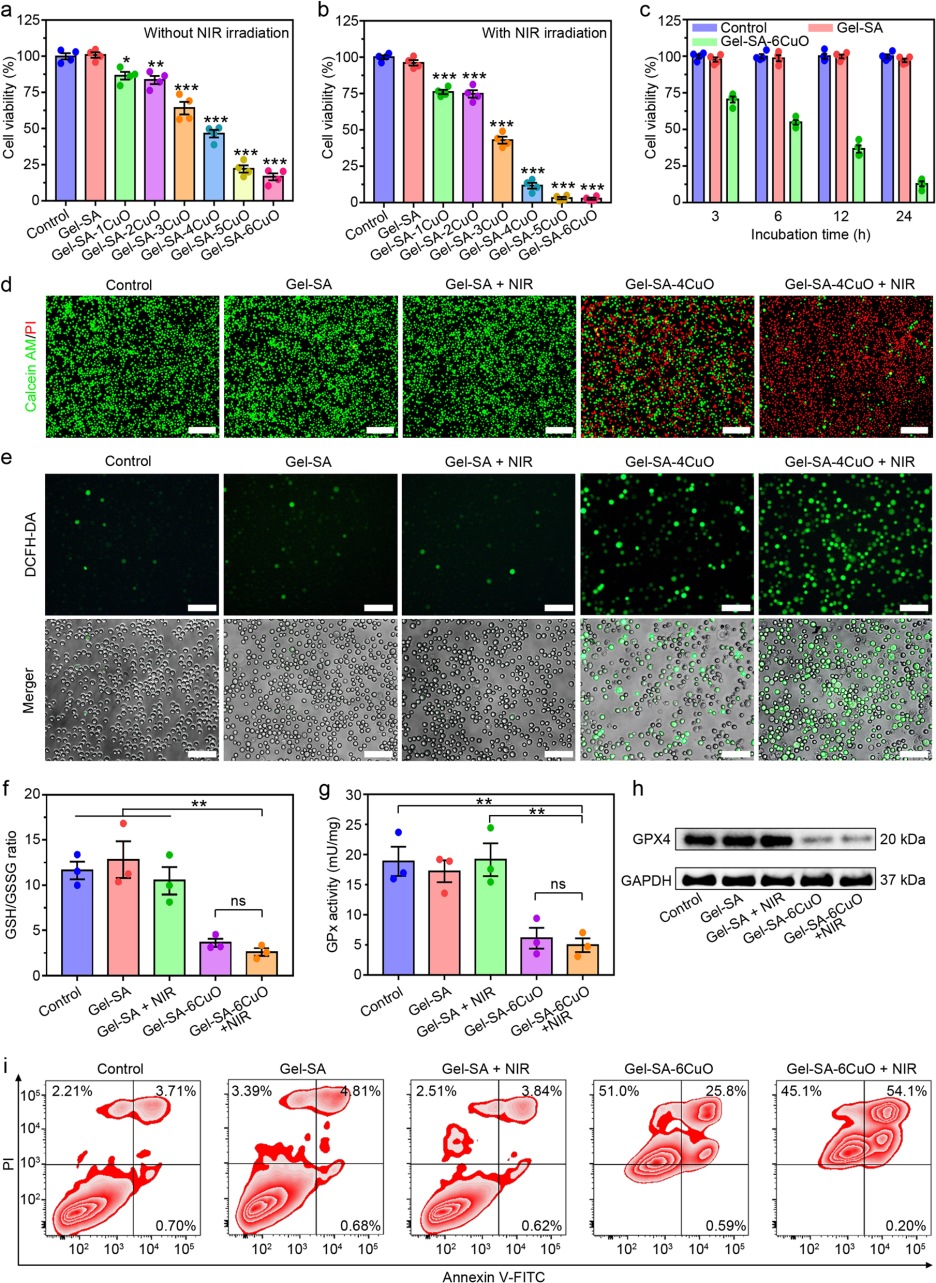
In vitro anticancer efficacy of Gel-SA-CuO hydrogel scaffolds. Viability of H22 cells treated with indicated scaffolds with (**a**) or without (**b**) NIR irradiation. **c** Effect of incubation time on the viability of H22 cells treated with indicated scaffolds. **d** Fluorescence images of H22 cells after various treatments and stained with calcein-AM and PI. Scale bars represent 200 μm. **e** Detection of intracellular ROS generation of H22 cells after various treatments and stained with DCFH-DA. Scale bars represent 100 μm. **f** Measurement of GSH/GSSG ratio in H22 cells with indicated treatments. **g** GPx activity of H22 cells with various treatments. **h** The GPX4 expression of H22 cells with various treatments. **i** Detection of cell apoptosis of H22 cells under indicated treatments

Regarding the good performance of ·OH generation and photothermal conversion efficiency, the anticancer effect of Gel-SA-CuO hydrogel scaffolds was evaluated in vitro. After hydrogel scaffolds were incubated with murine hepatocellular carcinoma H22 cells for 24 h, the viability of cells was determined by MTT assay. As shown in Fig. 6a, cell viability for Gel-SA group was similar to that of the control group, indicating that Gel-SA hydrogel scaffold had good biocompatibility. In contrast, cell viability decreased gradually from Gel-SA-1CuO to Gel-SA-6CuO scaffolds, which indicated that Gel-SA-CuO hydrogel scaffolds effectively eradicated H22 cells and the anticancer efficiency could be adjusted by CuO content in the hydrogel scaffolds. We further examined the effects of NIR exposed hydrogel scaffolds on hepatocellular carcinoma cells. While NIR alone or Gel-SA + NIR showed no noticeable impacts on H22 cells, NIR exposed Gel-SA-CuO hydrogel scaffolds significantly decreased the viability of H22 cells, indicating that the NIR potentially improved the therapeutic efficiency of Gel-SA-CuO hydrogel scaffolds (Fig. 6b). Additionally, it was observed that the incubation time significantly potentiated the effects of Gel-SA-6CuO hydrogel scaffold on killing H22 cells. The percentage of viable H22 cells decreased to 70.504%, 54.821%, 36.605%, 12.802% with Gel-SA-6CuO treatment for 3 h, 6 h, 12 h, and 24 h, respectively (Fig. 6c). In congruence with this, the calcein AM and propidium iodide (PI) double staining further confirmed that Gel-SA-CuO hydrogel scaffolds with NIR irradiation were most potent in inducing the death of H22 cells (Fig. 6d).

The role of CuO in cancer therapy has been reported through multiple mechanisms. For example, Zhang et al. reported that CuO could generate Cu(II) to induce oxidative stress, downregulate GSH levels, inactivate GPX4, and activate SOD-1, which showed a strong inhibition effect on tumor growth and recurrence [62]. Manisha et al. demonstrated CuO nanowire could lead to mitochondrial-dependent apoptosis through activating PTEN [71]. To decipher the underlying mechanism involved in the anticancer effects of Gel-SA-CuO hydrogel scaffolds, cells with different treatments were stained with 2′,7′-dichlorofluorescein diacetate (DCFH-DA) to evaluate the intracellular ROS levels. As displayed in Fig. 6e, few cells exhibited green fluorescence after treatment of Gel-SA hydrogel scaffold with or without NIR irradiation. In contrast, treatment with Gel-SA-4CuO hydrogel scaffold induced strong green fluorescence, which was attributed to the intracellular generation of ROS through Fenton-like reaction. Moreover, the green fluorescence was enhanced under the NIR irradiation, demonstrating that combining Gel-SA-4CuO hydrogel scaffold with laser irradiation led to much more ROS production. Consequently, the intracellular ratio of GSH/GSSG decreased after treatment of Gel-SA-6CuO hydrogel scaffold, which confirmed Cu^2+^ could be reduced to Cu^+^ by GSH (Fig. 6f). Considering the depletion of GSH could induce ferroptosis via the inactivation of GPX4 [72–74], we then investigated the activity of GPx based on the oxidation of reduced nicotinamide adenine dinucleotide phosphate (NADPH) to nicotinamide adenine dinucleotide phosphate (NADP). Remarkably, the activity of GPx significantly decreased in cells after the treatment of Gel-SA-6CuO hydrogel scaffold with or without laser irradiation (Fig. 6g). In addition, the GPX4 expression was dramatically downregulated by Gel-SA-6CuO and Gel-SA-6CuO + NIR, while the treatments of Gel-SA and Gel-SA + NIR exhibited a neglectable impact compared to the untreated group (Fig. 6h). These results indicated Gel-SA-6CuO could induce ferroptosis by GSH depletion-mediated inactivation of GPX4. Furthermore, cell apoptosis was analyzed by the Annexin V-FITC/PI double staining method. Consistent with the cell viability assays, the induction of apoptosis was significantly greater in cells treated by Gel-SA-6CuO and Gel-SA-6CuO + NIR than that of Gel-SA and Gel-SA + NIR (Fig. 6i). Altogether, the cell death pathway induced by Gel-SA-CuO hydrogel scaffolds involved both ferroptosis and apoptosis.

### In vivo therapeutic efficacy of Gel-SA-CuO hydrogel scaffolds

**Fig. 7:**
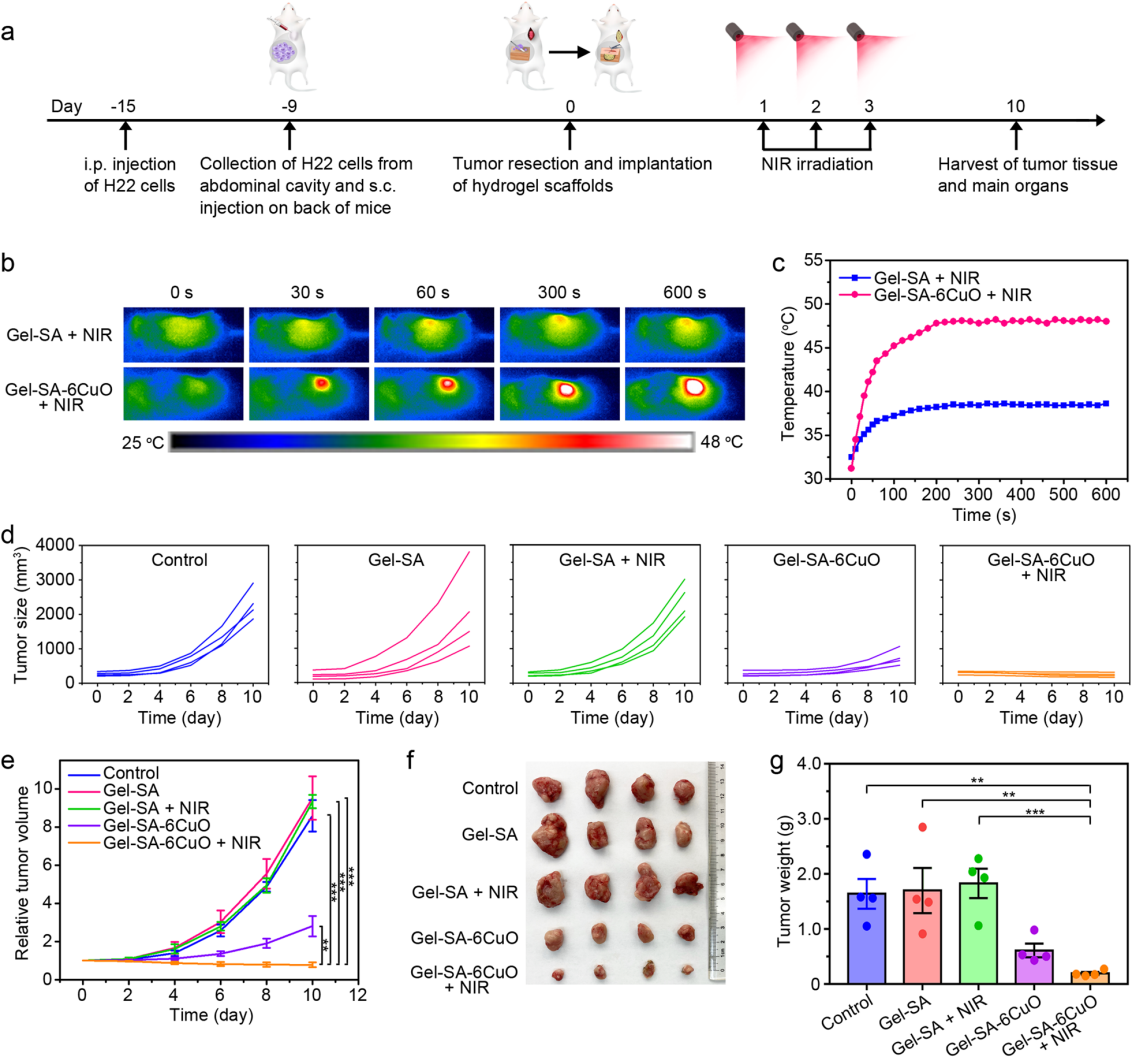
In vivo tumor therapeutic effect of Gel-SA-CuO hydrogel scaffolds. **a** Schematic illustration of the use of Gel-SA-CuO hydrogel scaffolds for inhibiting postsurgical recurrence. **b** Real-time thermal images of mice treated with Gel-SA hydrogel scaffolds and Gel-SA-6CuO hydrogel scaffolds under NIR irradiation. **c** Temperature curves in tumors of mice with indicated treatments over time. **d** Individual tumor growth curves of tumor-bearing mice after various treatments as indicated. **e** Relative tumor volume of mice with different treatments. **f** Photograph of tumors extracted from mice with different treatments on day 10. **g** Tumor mass of mice with indicated treatments

Encouraged by the good anticancer performance of Gel-SA-CuO hydrogel scaffolds in vitro, we further explored the therapeutic efficacy in vivo. Surgical resection models were utilized to evaluate the inhibition of cancer recurrence. After implanting Gel-SA-6CuO hydrogel scaffold in the tumor resection site, NIR irradiation was conducted (Fig. 7a). The temperature of mice treated with Gel-SA-6CuO + NIR increased and gradually came to around 48 °C (Fig. 7b and c). Comparatively, the temperature of mice treated with Gel-SA + NIR showed a slight increase to 38.6 °C. On day 10, all the tumor volume values in groups of control, Gel-SA, and Gel-SA + NIR exceeded 1000 mm^3^ (Fig. 7d). In contrast, the tumor volume values of four mice in Gel-SA-6CuO group were 1056.96 mm^3^, 512.91 mm^3^, 712.32 mm^3^, and 649.46 mm^3^, respectively, while that in Gel-SA-6CuO + NIR group were 167.58 mm^3^, 317.26 mm^3^, 258.51 mm^3^, and 212.62 mm^3^, respectively. The tumor volume after the treatment of Gel-SA-6CuO exhibited significant decrease than that of Gel-SA, which could be attributed to the GSH depletion induced ferroptosis and chemodynamic therapy induced apoptosis (Fig. 7e). Moreover, compared with Gel-SA-6CuO group, the Gel-SA-6CuO + NIR group inhibited the tumor growth more efficiently without tumor recurrence in the observation period, which could be ascribed to the synergistic PTT and PTT enhanced CDT. Consistent with the tumor volume, the tumor weight measured at the end of this study also showed a remarkable reduction in Gel-SA-6CuO and Gel-SA-6CuO + NIR groups (Fig. 7f and g).

**Fig. 8:**
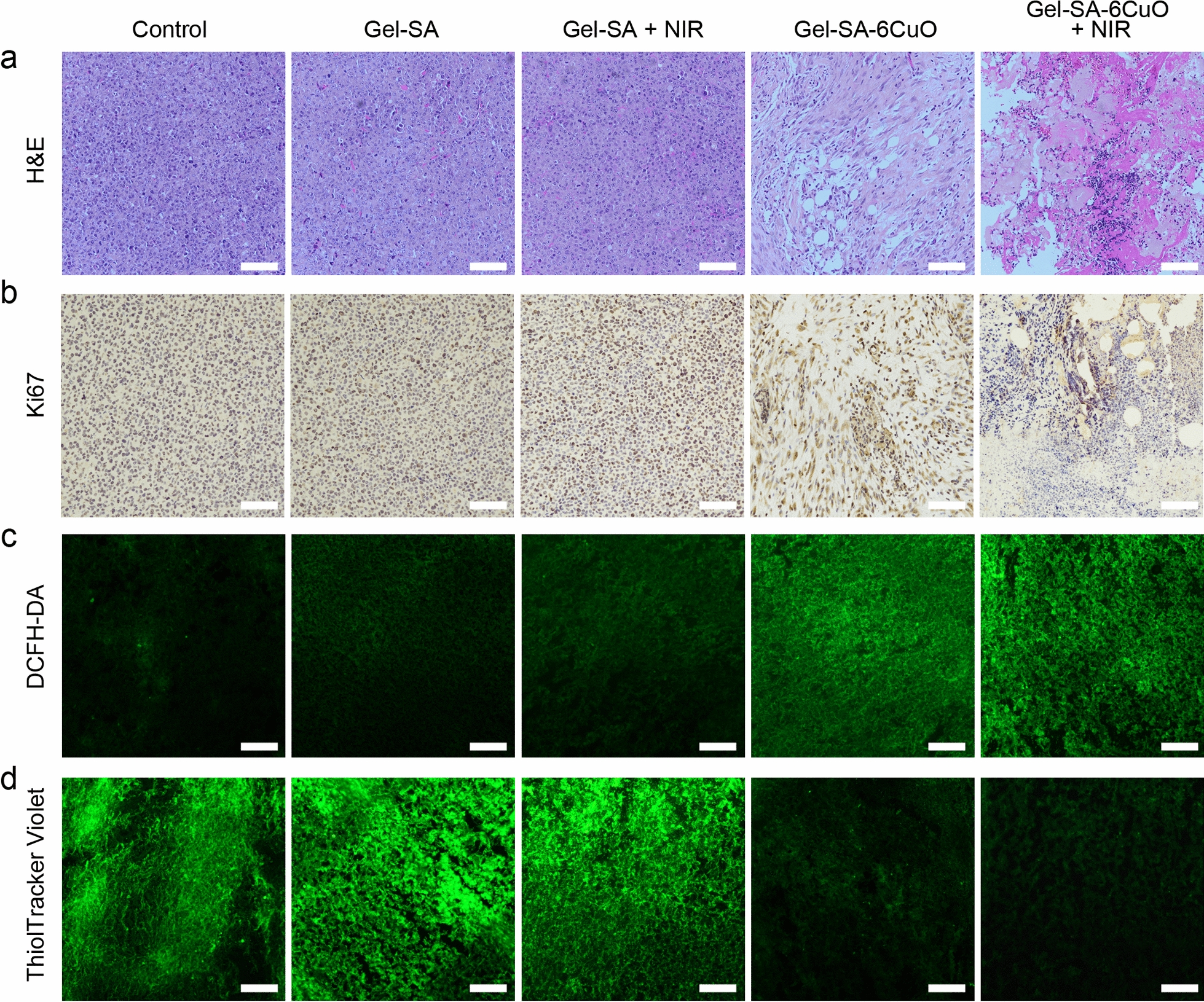
Histology and immunofluorescence analysis of tumor tissues. **a** H&E-stained tumor slices collected from mice after various treatments. **b** Ki-67 staining tumor slices extracted from mice with different treatments. **c** ROS staining in tumors extracted from mice with different treatments. **d** Intracellular GSH levels of tumors extracted from mice with indicated treatments. Scale bars represent 100 μm

To further confirm the antitumor efficacy of Gel-SA-CuO hydrogel scaffolds, histology and immunofluorescence analysis were conducted. As shown in Fig. 8a, hematoxylin and eosin (H&E) assay showed complete and compact structures in control, Gel-SA, and Gel-SA + NIR groups, whereas fragments and nuclear shrinkage were observed in Gel-SA-6CuO and Gel-SA-6CuO + NIR groups. In addition, Ki-67 staining assay was performed to assess cell proliferation. For control, Gel-SA, and Gel-SA + NIR groups, Ki-67 nucleoprotein overlapped with blue cell nucleus had high expression in tumor cells, indicating that these tumor cells had excellent proliferation ability (Fig. 8b). For Gel-SA-6CuO group, Ki-67 nucleoprotein decreased significantly. Especially, Ki-67 nucleoprotein expression in Gel-SA-6CuO + NIR group was rare and lots of aggregated blue cell nuclear were observed, which demonstrated Gel-SA-6CuO + NIR possessed the highest antitumor efficiency among all the groups. Furthermore, tumor tissues collected from Gel-SA-6CuO and Gel-SA-6CuO + NIR groups exhibited high ROS levels (Fig. 8c). Consistent with this result, the levels of GSH in tumor tissues in Gel-SA-6CuO and Gel-SA-6CuO + NIR groups greatly decreased, which indicated that the released Cu^2+^ from Gel-SA-6CuO hydrogel scaffold consumed the GSH to generate Cu^+^ for subsequent reaction with H_2_O_2_ to produce ROS via Fenton-like reaction (Fig. 8d). In addition, no obvious fluctuation of bodyweight of mice of all groups was observed (Additional file 1: Fig. S8). Moreover, H&E staining of major organs including heart, liver, spleen, lung, and kidney showed no noticeable lesions or inflammatory, indicating good biocompatibility of Gel-SA-6CuO hydrogel scaffold (Additional file 1: Fig. S9). Collectively, all the results suggest that Gel-SA-CuO hydrogel scaffolds serve as efficient therapeutic implants to inhibit postoperative tumor recurrence.

## Conclusion

In summary, we have developed 3D printed hydrogel scaffolds with combined photothermal and chemodynamic therapy for inhibiting postoperative tumor recurrence. The concentration of CuO can be flexibly adjusted in the hydrogel scaffolds. Notably, the released CuO nanoparticles from the hydrogel scaffolds can not only function as reservoir for releasing Cu^2+^ to produce intracellular ROS but also serve as photothermal agents to generate heat and thus further improve the efficiency of Fenton-like reaction. Moreover, GSH depletion by the released Cu^2+^ from the hydrogel scaffolds induces ferroptosis via the inactivation of GPX4. More importantly, inhibition of tumor recurrence after primary resection can be achieved by implanting hydrogel scaffolds in the resection site. Therefore, this study may provide new insight into the design of the multifunctional implantable devices for diagnosis, disease monitoring, and therapy.

## Supplementary Information

**Additional file 1: Figure S1.** Top-view, front-view and side-view pictures of Gel-SA, Gel-SA-4CuO, and Gel-SA-6CuO hydrogel scaffolds. **Figure S2.** Digital photographs of Gel-SA scaffold, Gel-SA-1CuO scaffold, Gel-SA-2CuO scaffold, Gel-SA-3CuO scaffold, Gel-SA-4CuO scaffold, Gel-SA-5CuO scaffold, and Gel-SA-6CuO scaffold under dry conditions from left to right. **Figure S3.** High-resolution XPS spectra of Cu 2p for CuO nanoparticles. **Figure S4.** Representative photographs of swelling behavior of Gel-SA-4CuO hydrogel scaffold. **Figure S5.** The side view of Gel-SA and Gel-SA-6CuO hydrogel scaffolds after incubation with water for indicated time. **Figure S6.** Representative photos of Gel-SA and Gel-SA-6CuO hydrogel scaffolds after degradation for 24 h. **Figure S7.** Time-dependent release of Cu^2+^ from Gel-SA-CuO scaffolds. **Figure S8.** Changes of body weight of mice with different treatments. **Figure S9.** The representative H&E staining images of major organs from tumor-bearing mice with different treatments.

## Data Availability

All data generated and analyzed during this study are included in the manuscript and Additional file 1.
