# Supplementary material for: 3D printed hydrogel scaffolds combining glutathione depletion-induced ferroptosis and photothermia-augmented chemodynamic therapy for efficiently inhibiting postoperative tumor recurrence
Source: J Nanobiotechnology. 2022 Jun 7;20:266. doi: 10.1186/s12951-022-01454-1 (PMC9171966; doi:10.1186/s12951-022-01454-1)
Supplement: Supplementary file 1 — Additional file 1: Figure S1. Top-view, front-view and side-view pictures of Gel-SA, Gel-SA-4CuO, and Gel-SA-6CuO hydrogel scaffolds. Figure S2. Digital photographs of Gel-SA scaffold, Gel-SA-1CuO scaffold, Gel-SA-2CuO scaffold, Gel-SA-3CuO scaffold, Gel-SA-4CuO scaffold, Gel-SA-5CuO scaffold, and Gel-SA-6CuO scaffold under dry conditions from left to right. Figure S3. High-resolution XPS spectra of Cu 2p for CuO nanoparticles. Figure S4. Representative photographs of swelling behavior of Gel-SA-4CuO hydrogel scaffold. Figure S5. The side view of Gel-SA and Gel-SA-6CuO hydrogel scaffolds after incubation with water for indicated time. Figure S6. Representative photos of Gel-SA and Gel-SA-6CuO hydrogel scaffolds after degradation for 24 h. Figure S7. Time-dependent release of Cu2+ from Gel-SA-CuO scaffolds. Figure S8. Changes of body weight of mice with different treatments. Figure S9. The representative H&E staining images of major organs from tumor-bearing mice with different treatments. [file 12951_2022_1454_MOESM1_ESM.docx]

3D printed hydrogel scaffolds combining glutathione depletion-induced ferroptosis and photothermia-augmented chemodynamic therapy for efficiently inhibiting postoperative tumor recurrence

Wentao Dang^1,#^, Wei-Chih Chen^1,2,#^, Enguo Ju^1,*,#^, Yanteng Xu^1^, Kai Li^3^, Haixia Wang^1^, Kun Wang^2, *^, Shixian Lv^4^, Dan Shao^5^, Yu Tao^1,6,*^, and Mingqiang Li^1,6,*^

1. Laboratory of Biomaterials and Translational Medicine, Center for Nanomedicine, The Third Affiliated Hospital, Sun Yat-sen University, Guangzhou 510630, China

2. Department of Joint and Trauma Surgery, The Third Affiliated Hospital, Sun Yat-sen University, Guangzhou 510630, China

3. Department of Ultrasound, The Third Affiliated Hospital, Sun Yat-sen University, Guangzhou 510630, China

4. School of Materials Science and Engineering, Peking University, Beijing 100871, China

5. Institutes of Life Sciences, School of Biomedical Sciences and Engineering, South China University of Technology, Guangzhou 510006, China

6. Guangdong Provincial Key Laboratory of Liver Disease Research, Guangzhou 510630, China

Corresponding authors: E-mail:

jueng@mail.sysu.edu.cn (Enguo Ju), wangk@mail.sysu.edu.cn (Kun Wang), limq567@mail.sysu.edu.cn (Mingqiang Li), taoy28@mail.sysu.edu.cn (Yu Tao)

# Equal contributions.


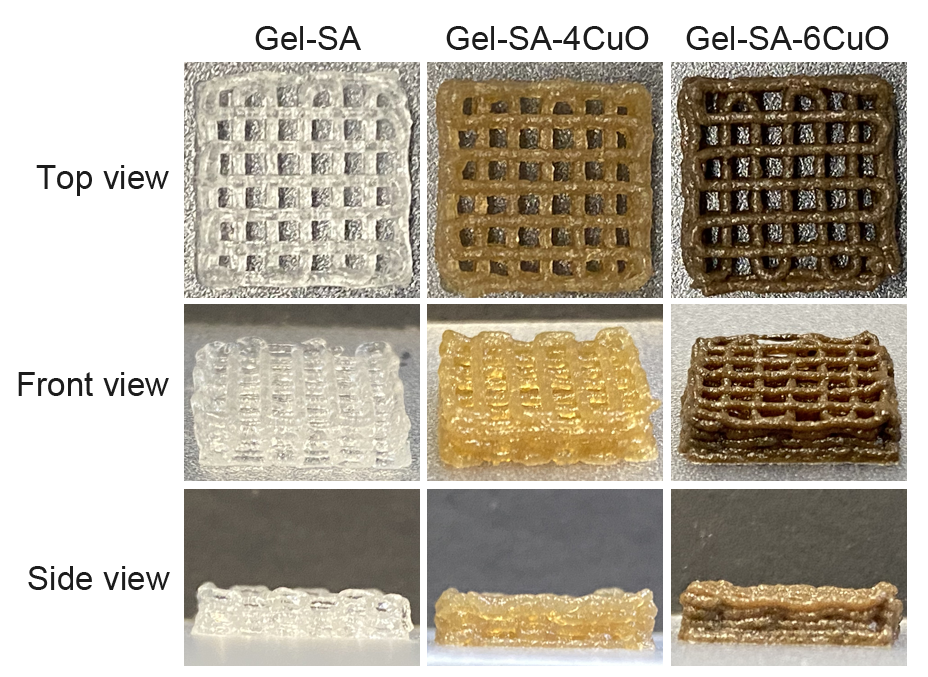


**Figure S1**. Top-view, front-view and side-view pictures of Gel-SA, Gel-SA-4CuO, and Gel-SA-6CuO hydrogel scaffolds.


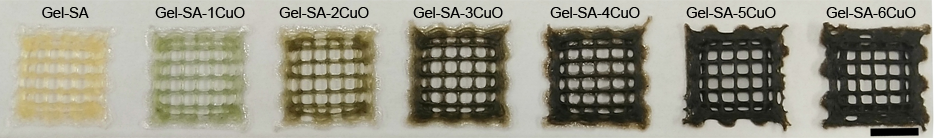


**Figure S2**. Digital photographs of Gel-SA scaffold, Gel-SA-1CuO scaffold, Gel-SA-2CuO scaffold, Gel-SA-3CuO scaffold, Gel-SA-4CuO scaffold, Gel-SA-5CuO scaffold, and Gel-SA-6CuO scaffold under dry conditions from left to right. Scale bar represents 5 mm.


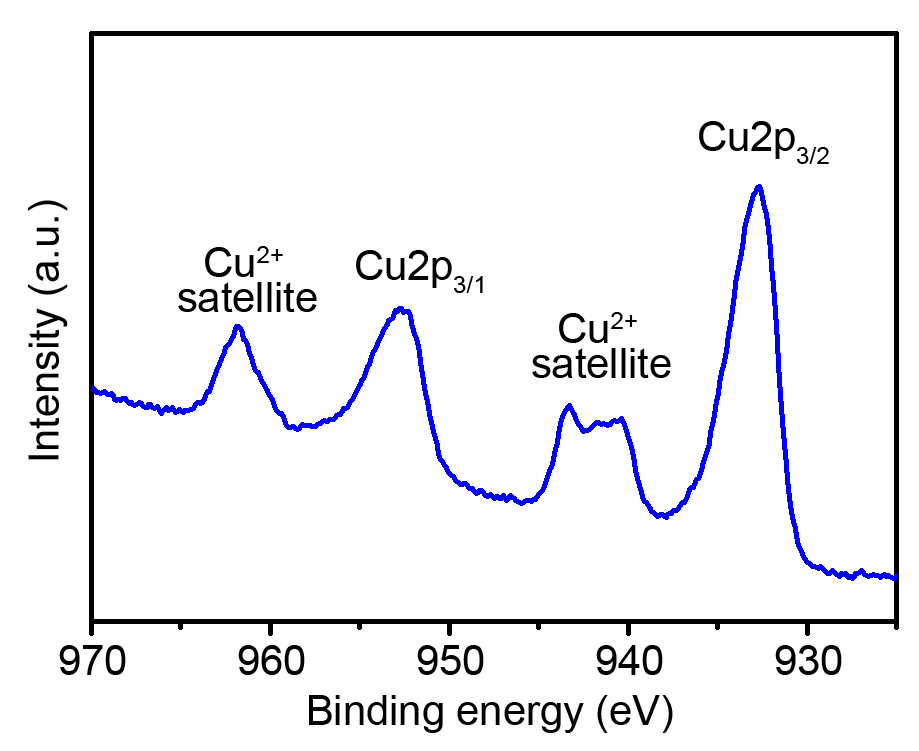


**Figure S3**. High-resolution XPS spectra of Cu 2p for CuO nanoparticles.


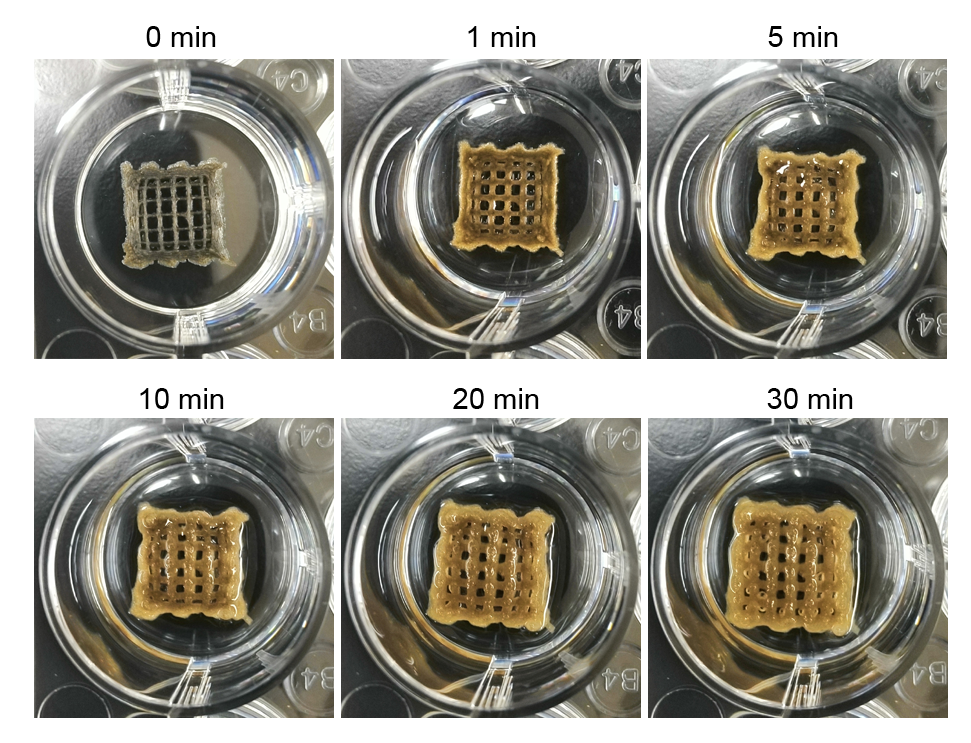


**Figure S4**. Representative photographs of swelling behavior of Gel-SA-4CuO hydrogel scaffold.


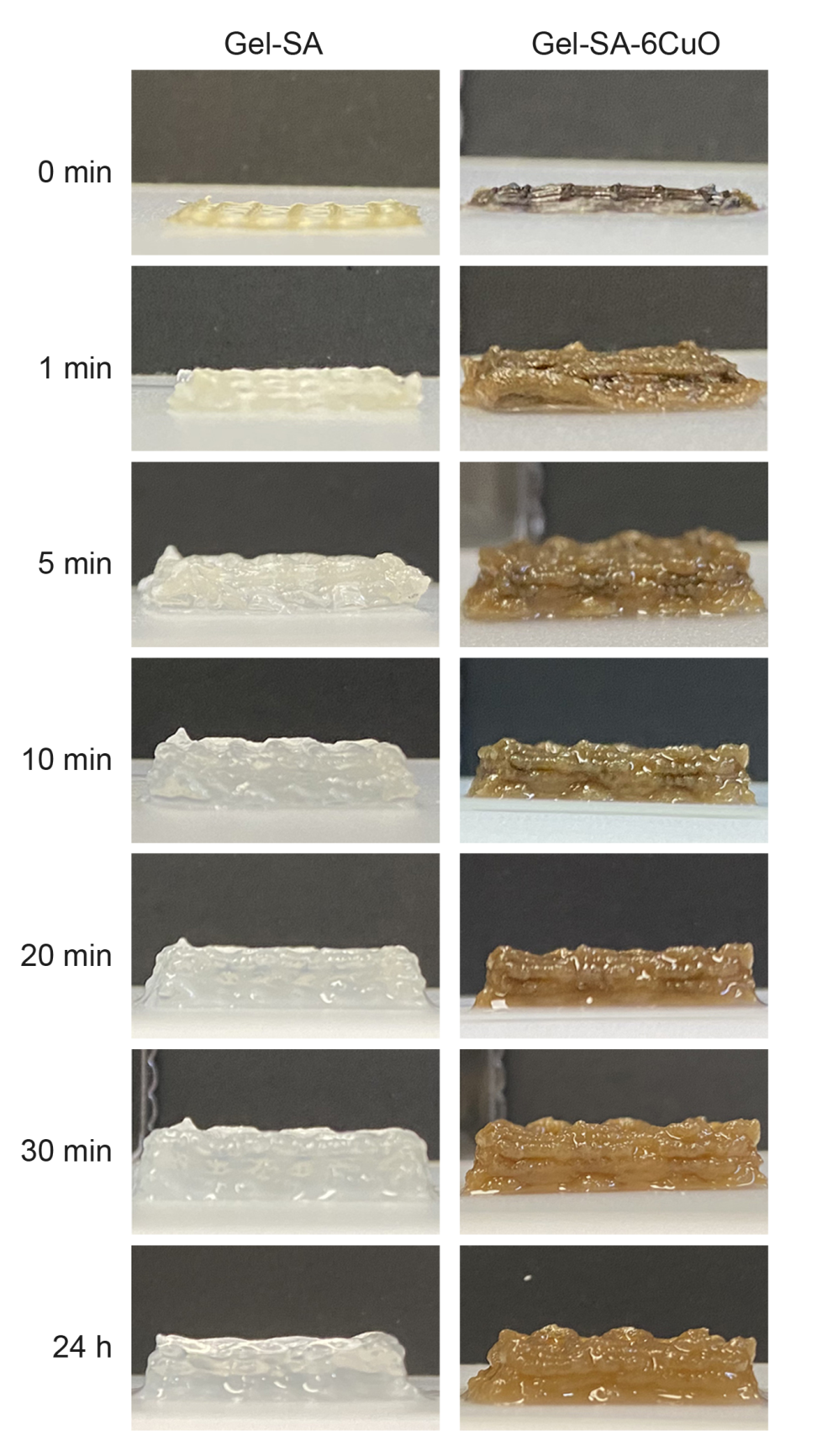


**Figure S5**. The side view of Gel-SA and Gel-SA-6CuO hydrogel scaffolds after incubation with water for indicated time.


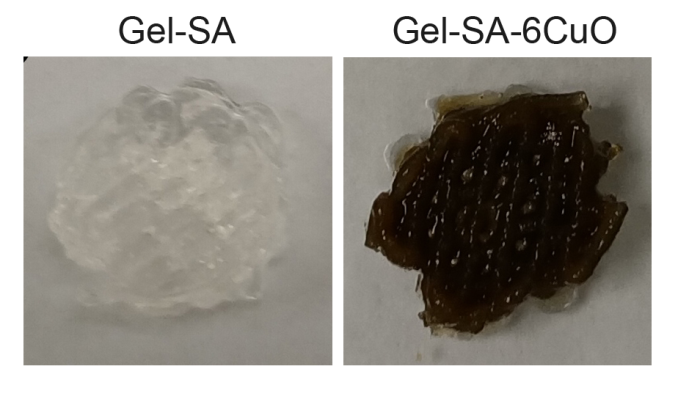


**Figure S6.** Representative photos of Gel-SA and Gel-SA-6CuO hydrogel scaffolds after degradation for 24 h.


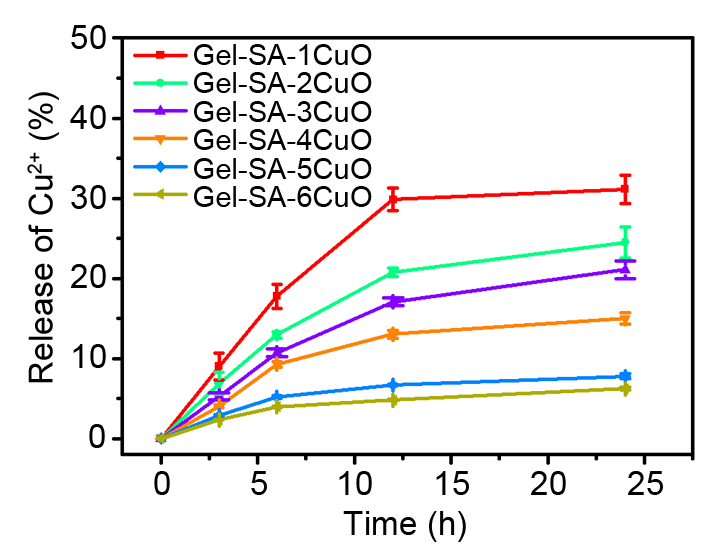


**Figure S7**. Time-dependent release of Cu^2+^ from Gel-SA-CuO scaffolds.


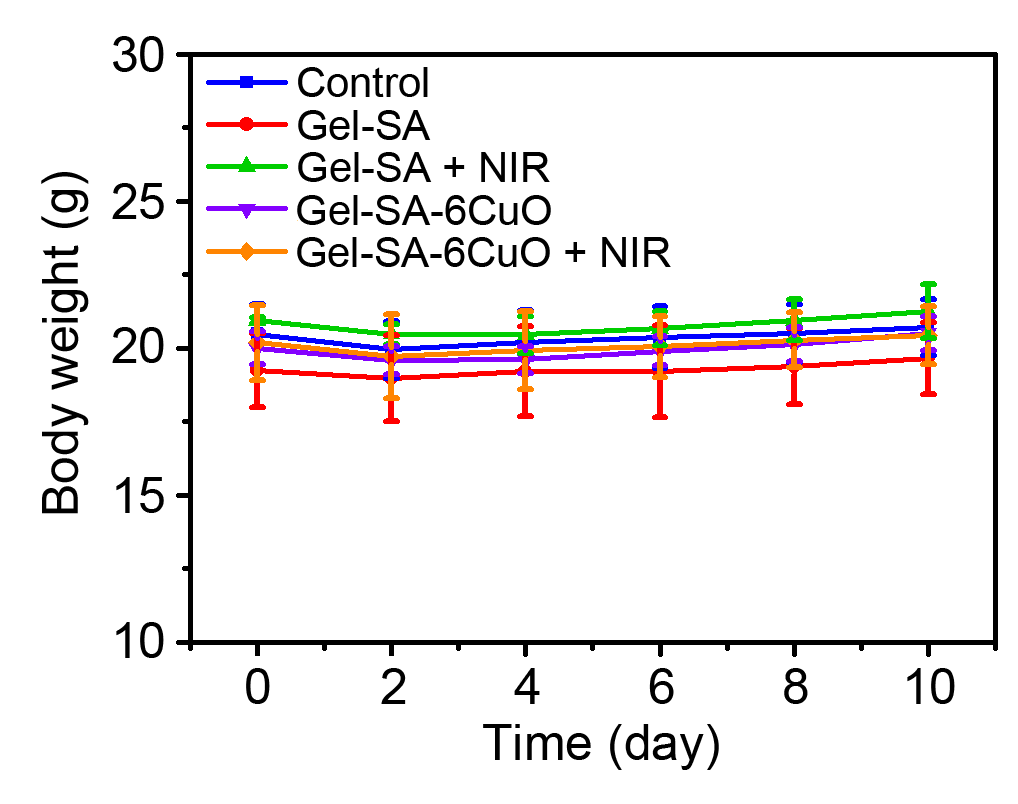


**Figure S8**. Changes of body weight of mice with different treatments.


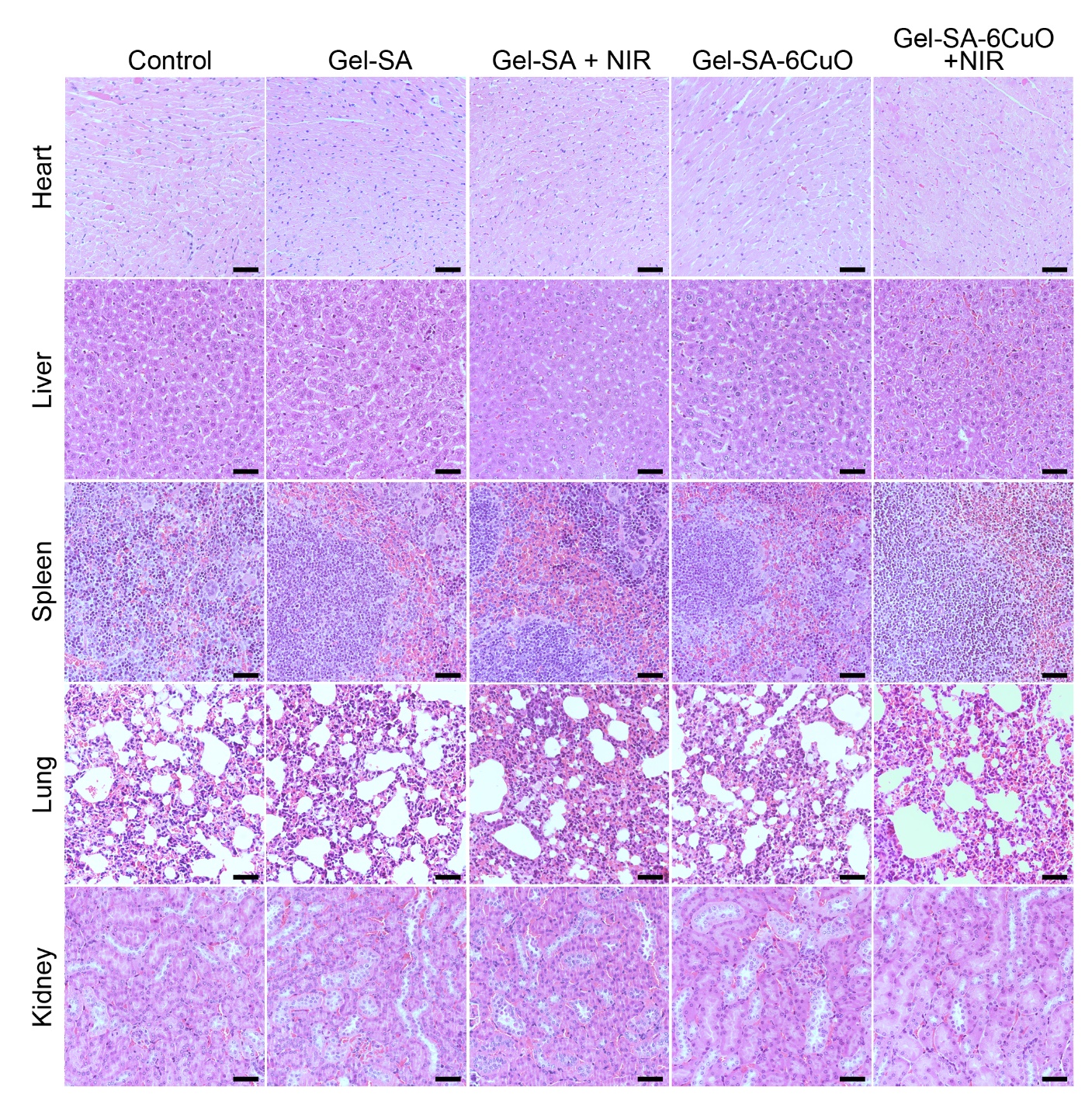


**Figure S9**. The representative H&E staining images of major organs from tumor-bearing mice with different treatments. Scale bars represent 50 μm.
